# Supplementary material for: The ongoing invasion of the endogenous retrovirus Kuruka in natural Drosophila melanogaster populations
Source: Nat Commun. 2026 Sep 16;17:10789. doi: 10.1038/s41467-026-77761-0 (PMC13669377; doi:10.1038/s41467-026-77761-0)
Supplement: Supplementary file 1 [file 41467_2026_77761_MOESM1_ESM.pdf]

Supplementary Figures

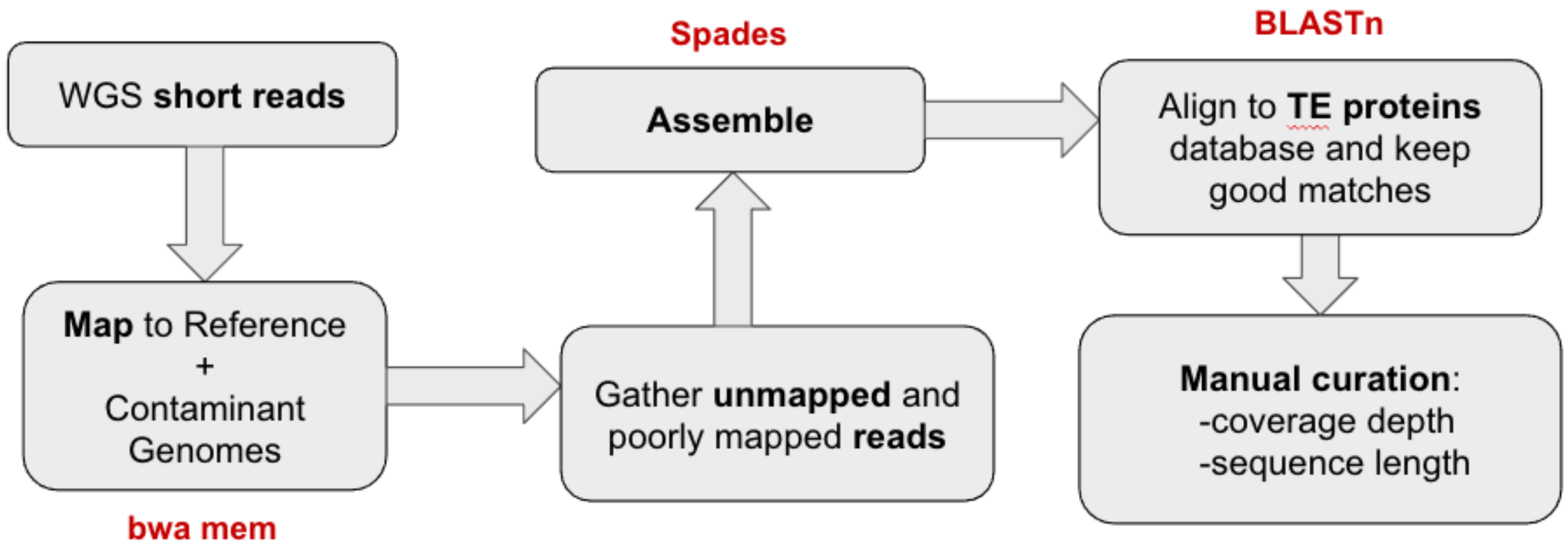

Supplementary Figure 1: Schematic of the pipeline used to identify potential non-reference TE families, like *Kuruka*. Whole genome sequencing short reads from various *D. melanogaster* populations were mapped to the reference genome and to potential contaminant genomes. The unmapped and poorly mapped reads were gathered and assembled. The resulting contigs were aligned to a TE protein database to filter for TE related sequences. A final step of manual curation taking into consideration metrics such as coverage depth and sequence length was necessary to filter outliers. In the end, only *Kuruka* emerged as new TE from this pipeline.

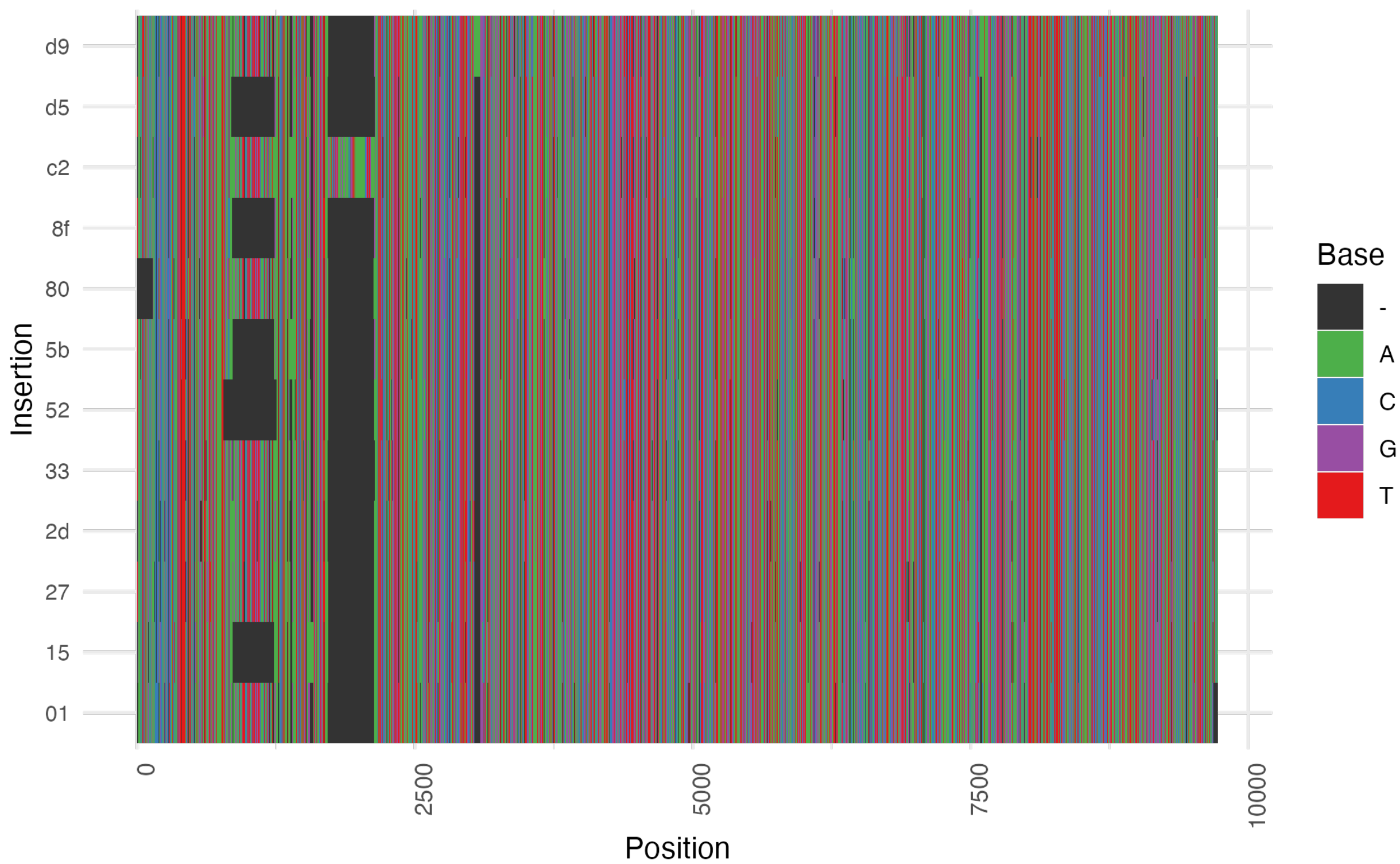

Supplementary Figure 2: Multiple sequence alignment of the 12 *Kuruka* insertions found in the long reads sequenced from the *D. melanogaster* samples collected in 2023 in North Dakota. The colors represent the 4 bases and alignment gaps. A common structural variant is evident around position 1000, where 7 insertions have the variant and the other 5 only have a big gap.

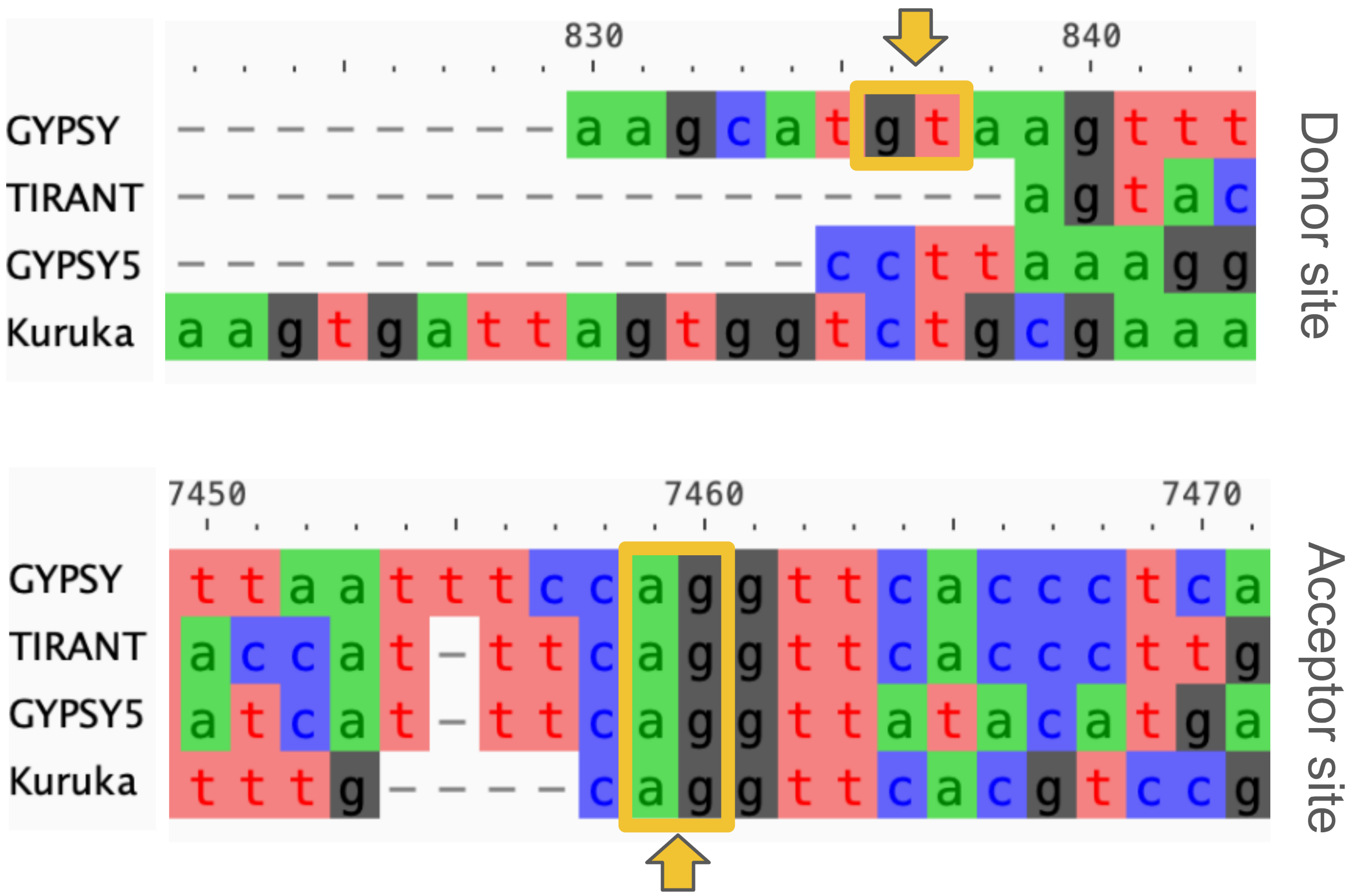

Supplementary Figure 3: Multiple sequence alignment of errantivirus sequences in the region of the env splice sites. Alignments are shown for two regions of the multiple sequence alignment of *Gypsy*, *Tirant*, *Gypsy5*, and *Kuruka*, corresponding to the annotated splice donor (top) and splice acceptor (bottom) sites of Gypsy[1]. The experimentally determined splice sites in *Gypsy* are highlighted with orange boxes. Alignment positions are numbered according to the multiple sequence alignment coordinates. The splice donor dinucleotide is not conserved across elements (or not aligned), whereas the splice acceptor (AG, bottom) is perfectly conserved in all four sequences, suggesting that env expression from a spliced subgenomic RNA is a shared feature of these errantiviruses.

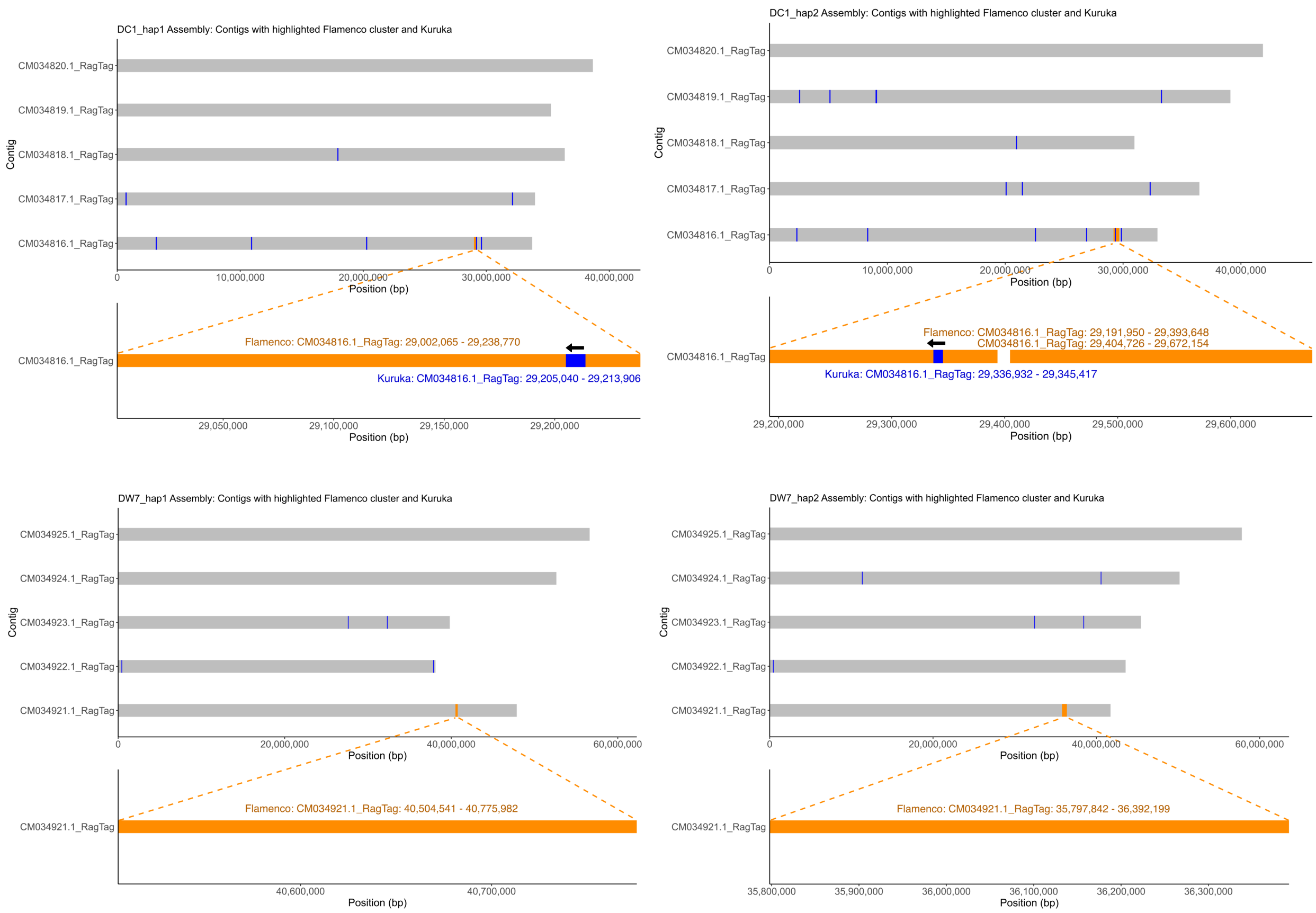

Supplementary Figure 4: Position of *Kuruka* insertions (blue) in the long-read assemblies of *D. melanogaster* strains collected 2025 in North Dakota (DW) and Washington DC (DC). The position of the piRNA cluster *flamenco* is highlighted in yellow and two haplotypes are shown for each strain (hap1, hap2).

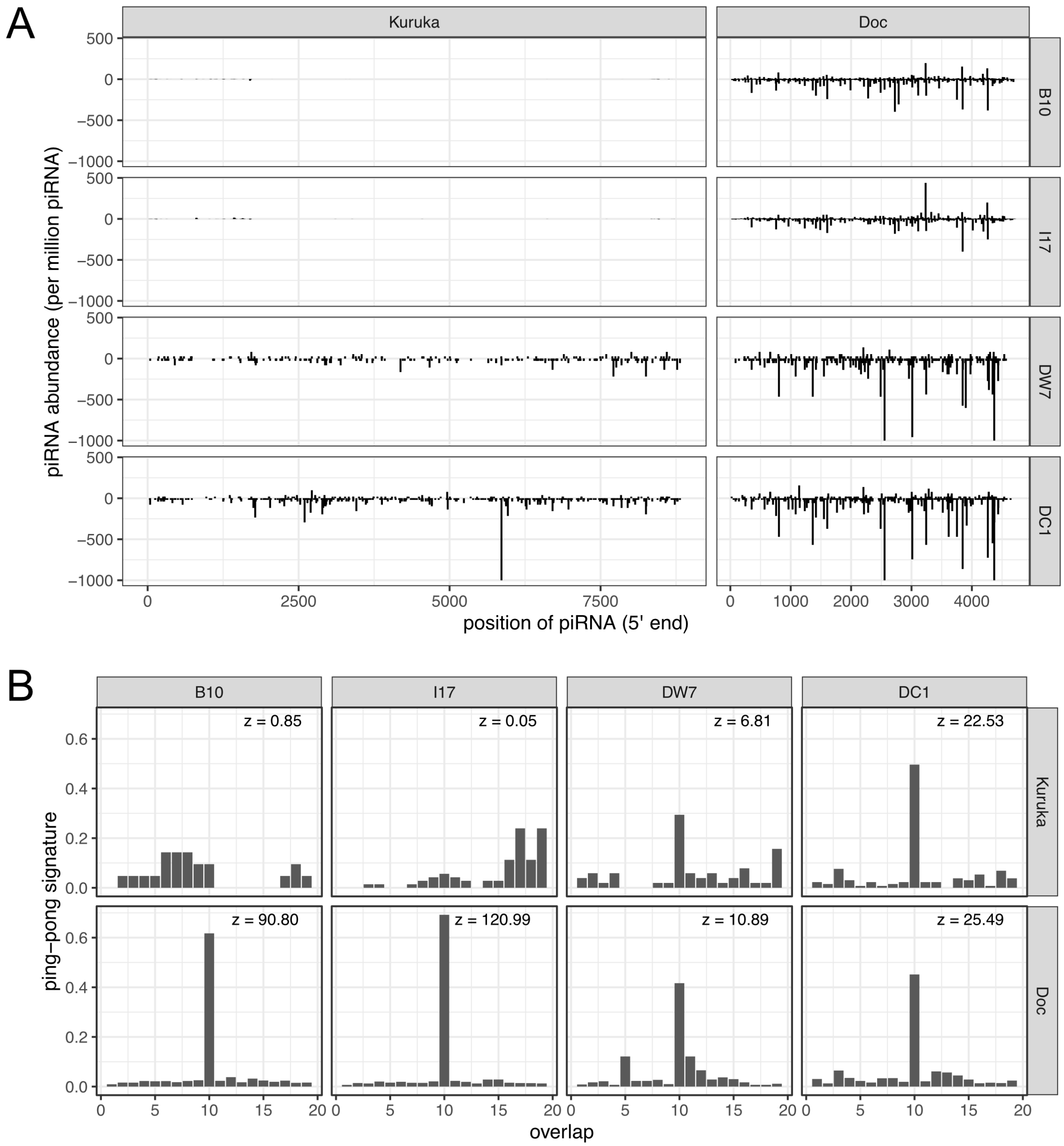

Supplementary Figure 5: Abundance of piRNAs for *Kuruka* and *Doc* in *D. melanogaster* strains collected at different years and geographic regions (I17 Ithaca, USA 2004; B10 Bejing, China 1995; DC1 Washington DC, USA, 2025; DW7 North Dakota, USA, 2025). A) Distribution of piRNAs along the TE sequence. Sense piRNAs are shown on the positive y-axis and antisense piRNAs on the negative y-axis B) *Kuruka* shows a ping-pong signature in DW7 and DC1. Ping-pong signatures for somatic TEs, such as the related *Tirant*, were observed before[2].

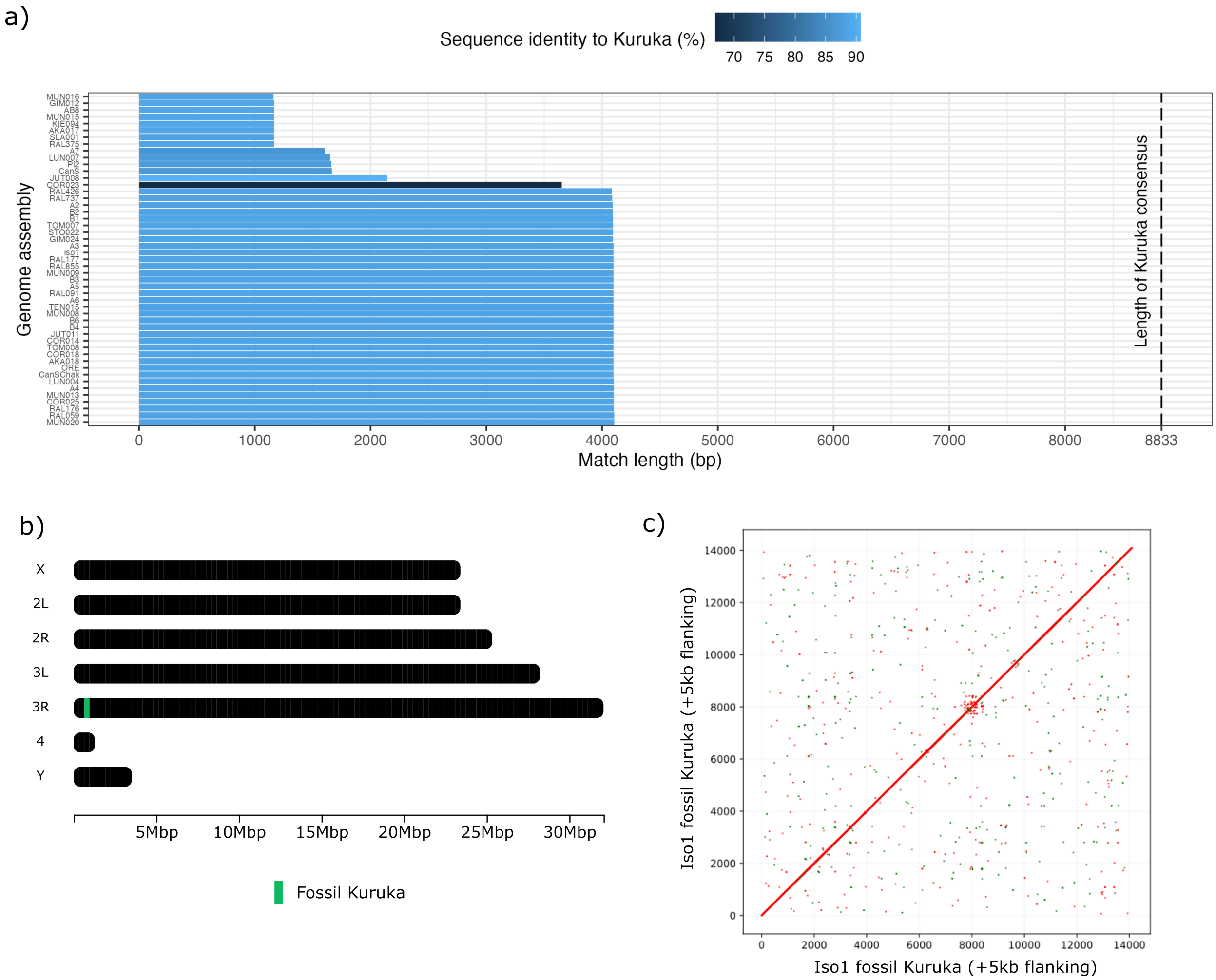

Supplementary Figure 6: a) Best *Kuruka* hits identified in 49 *D. melanogaster* genome assemblies, including the reference genome (Iso-1). A ∼4 kb sequence sharing ∼88% identity with *Kuruka* is found in 35 genomes; remaining assemblies contain only lower-similarity fragments. This sequence is referred to as the "fossil" *Kuruka*. b) The fossil *Kuruka* is consistently located at the distal end of chromosome arm 3R, inserted at the same genomic position in all 35 genomes where it is detected. c) No LTRs are detected in the fossil *Kuruka* sequence, even after examining 5 kb of flanking regions on both sides.

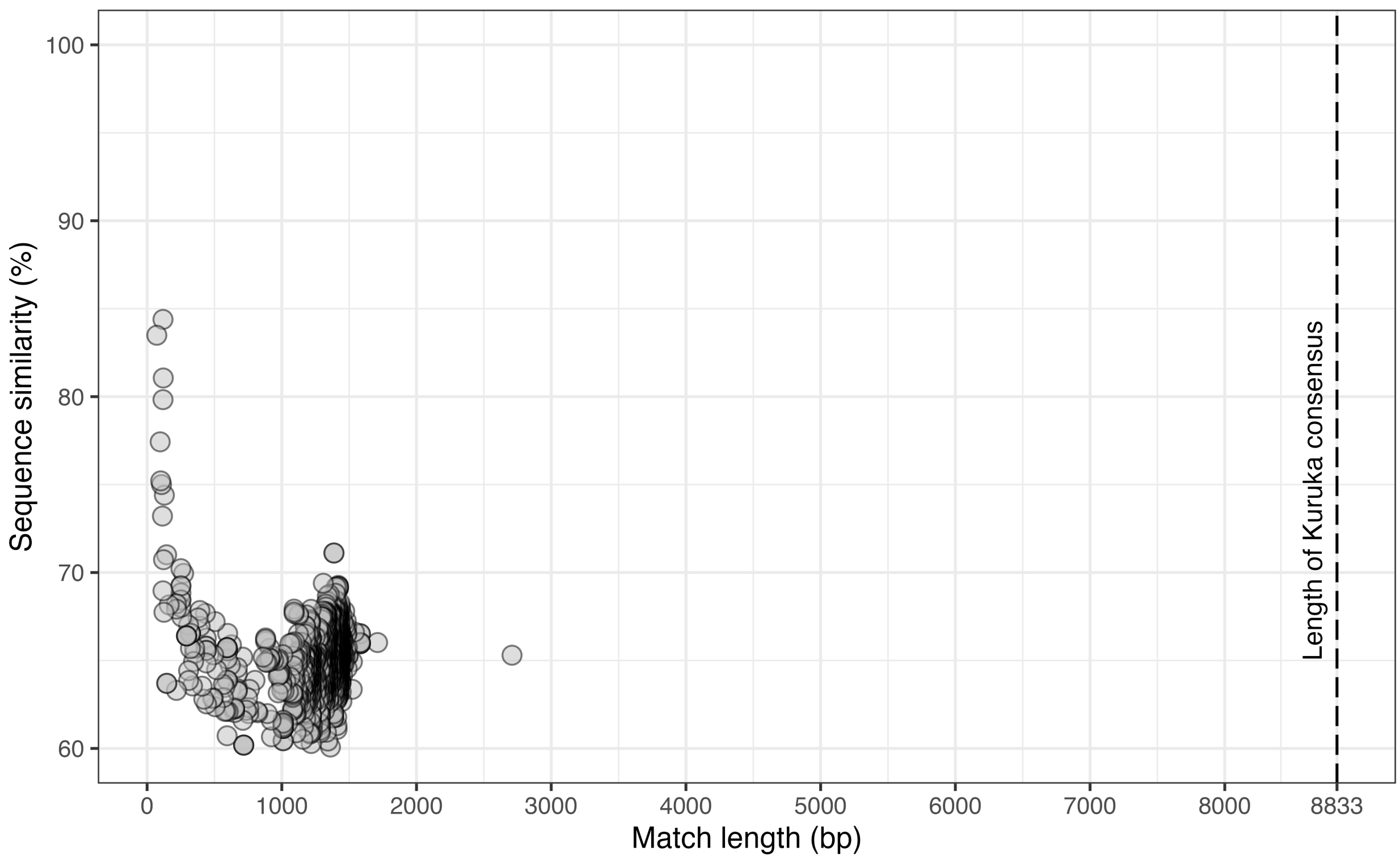

Supplementary Figure 7: Best *Kuruka* hits identified in 1211 non-drosophilids arthropods genome assemblies. The consensus sequence of *Kuruka* was used to search for similar sequences across the whole dataset, and the best match per genome is shown here. Only very small sequences (¡100bp) show a similarity ¿80% to *Kuruka*, so we conclude that no relevant match to any of the genomes can be highlighted.

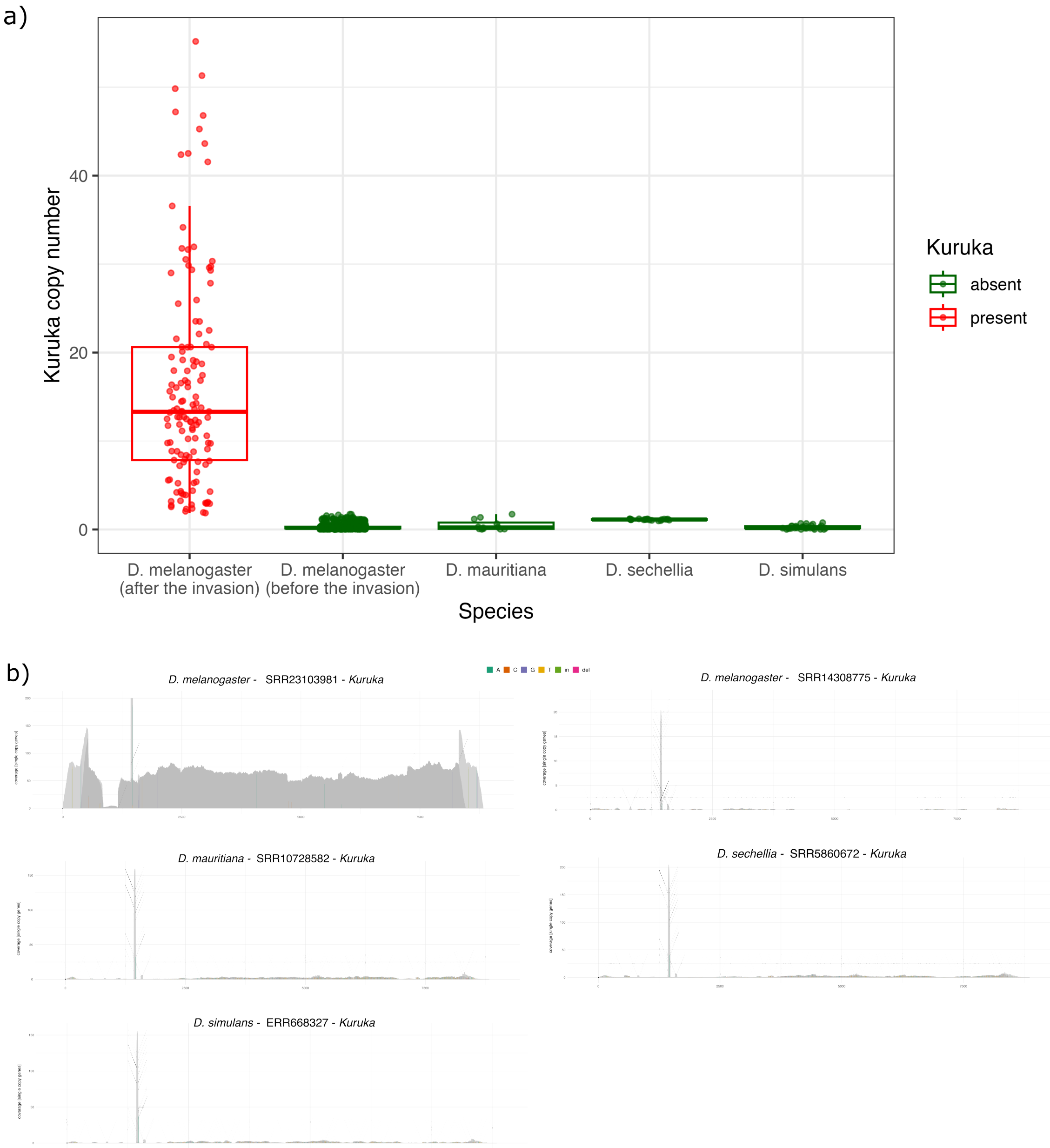

Supplementary Figure 8: *Kuruka* is absent in the species of the simulans clade. a) Estimated *Kuruka* copy number in 29 *D. simulans*, 21 *D. sechellia*, and 12 *D. mauritiana* samples. For comparison, *D. melanogaster* samples are included and separated into those collected before and after the *Kuruka* invasion. b) Representative sashimi plots showing short-read normalised coverage across the *Kuruka* consensus sequence. In the post-invasion *D. melanogaster* sample (first plot), reads map uniformly across the consensus with very few SNPs. In contrast, in all simulans-clade samples and in the pre-invasion *D. melanogaster* sample, reads predominantly map to a low-complexity region around 1300 bp, while the remainder of the sequence is covered only by a small number of reads containing numerous SNPs relative to the consensus (vertical colored lines).

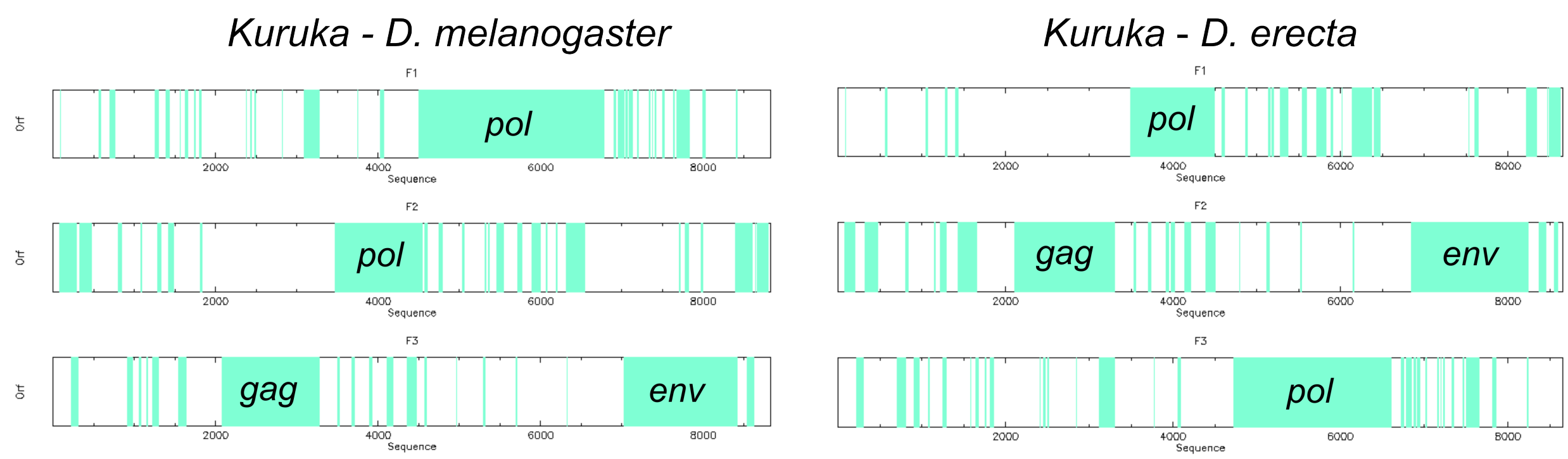

Supplementary Figure 9: Open reading frames found in the consensus sequence of *Kuruka* in *D. melanogaster* and in the *Kuruka* insertion in *D. erecta*. The three forward frames are shown. In both species, the *gag* and *env* proteins are found in the same frame, and the *pol* polyprotein is found splitted into two ORFs and in two distinct frames. The order of the ORFs in identical in the two species.

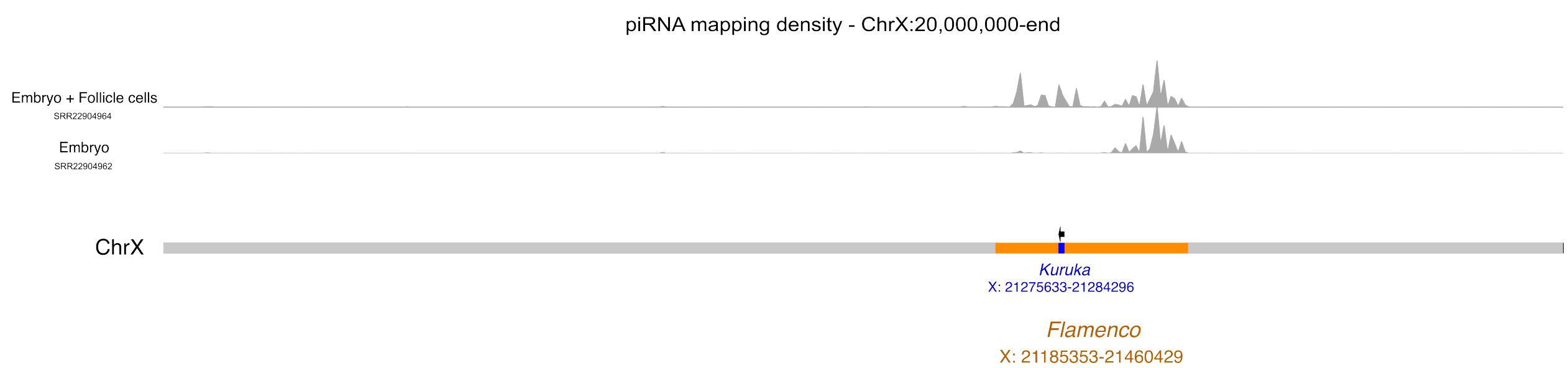

Supplementary Figure 10: Coverage of uniquely mapped piRNAs on the chromosome X's region containing *flamenco*, for the embryonic piRNAs sample and for one of the mixed piRNAs samples. In both, a hotspot of piRNAs corresponding to the *flamenco* annotation is present. Interestingly, the right part of *flamenco* seems to be producing piRNAs also in the embryo sample. The *Kuruka* insertion lies in the left part of the cluster, purely somatically expressed as it is absent in the embryo piRNAs. The insertion is antisense (see black arrow).

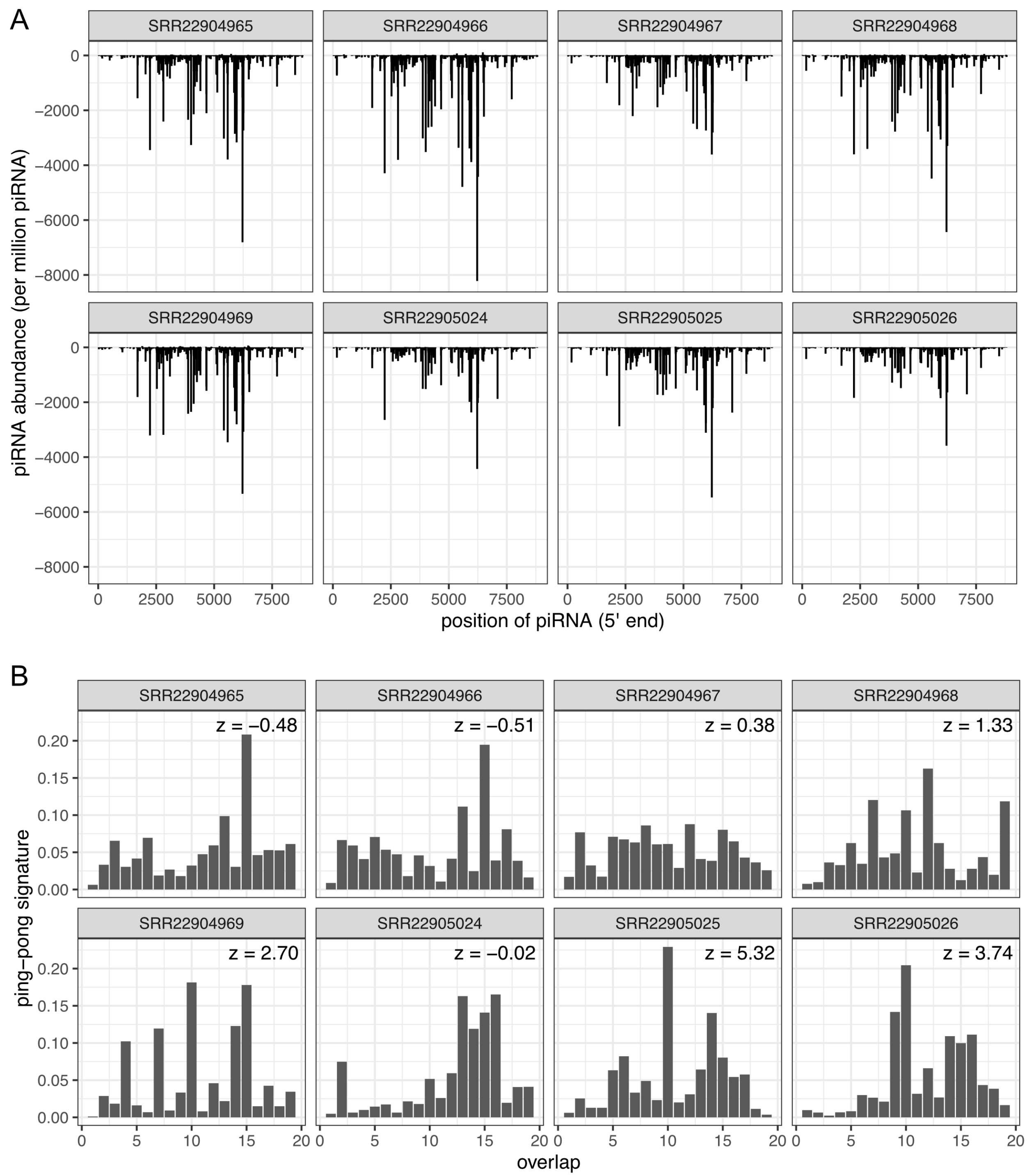

Supplementary Figure 11: A) piRNA distribution across the *Kuruka* sequence for samples not shown in the main figure. All of samples contain both germline and somatic cells. piRNAs complementary to *Kuruka* are consistently present in all samples. B) ping-pong signature for *Kuruka* in the same samples.

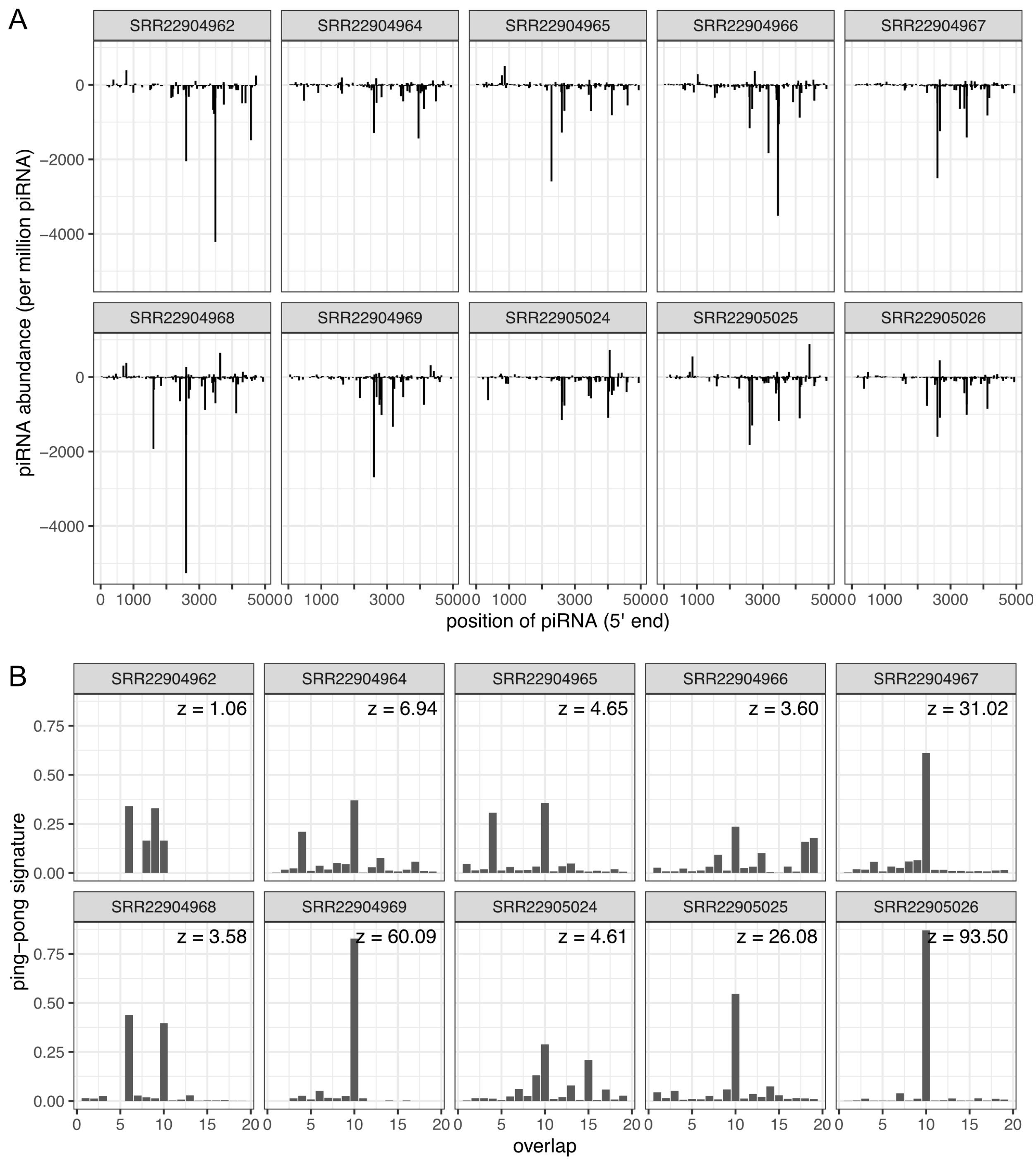

Supplementary Figure 12: A) piRNA distribution across the *jockey33* sequence in *D. erecta*. piRNAs are consistently mapping to the TE in all samples. B) ping-pong signature of *jockey33* in the same samples.

Supplementary Tables

Supplementary Table 1: The newly assembled *D. erecta* genome has less contigs but higher N50 and N90 compared to the reference genome. BUSCO found the same percentage of orthologous genes in both the assemblies.

	**GCA_003286155.2 (reference genome)**	**GCA_xxx (newly assembled genome)**
Contigs	94	49
N50 (bp)	28426653	30625941
N90 (bp)	778323	1614603
Complete BUSCOs (%)	99.7	99.7

References

[1] A. Pelisson, S. Song, N. Prud'Homme, P. Smith, A. Bucheton, and V. Corces. Gypsy transposition correlates with the production of a retroviral envelope-like protein under the tissue-specific control of the drosophila flamenco gene. *The EMBO journal*, 13(18):4401–4411, 1994.

[2] B. Rafanel, L. Protsenko, D. Handler, J. Brennecke, and K.-A. Senti. Retrovirus insertions in host transcripts trigger de novo piRNA immunity. *The EMBO Journal*, pages 1–26, May 2026. ISSN 1460-2075. doi: 10.1038/s44318-026-00777-1.
