# Supplementary material for: The ongoing invasion of the endogenous retrovirus Kuruka in natural Drosophila melanogaster populations
Source: Nat Commun. 2026 Sep 16;17:10789. doi: 10.1038/s41467-026-77761-0 (PMC13669377; doi:10.1038/s41467-026-77761-0)
Supplement: Supplementary file 2 [file 41467_2026_77761_MOESM2_ESM.pdf]

nature portfolio

Corresponding author(s): Riccardo Pianezza

Last updated by author(s): Aug 5, 2026

Reporting Summary

Nature Portfolio wishes to improve the reproducibility of the work that we publish. This form provides structure for consistency and transparency in reporting. For further information on Nature Portfolio policies, see our Editorial Policies and the Editorial Policy Checklist.

Statistics

For all statistical analyses, confirm that the following items are present in the figure legend, table legend, main text, or Methods section.

n/a	Confirmed	
☐	☒	The exact sample size (*n*) for each experimental group/condition, given as a discrete number and unit of measurement
☒	☐	A statement on whether measurements were taken from distinct samples or whether the same sample was measured repeatedly
☒	☐	The statistical test(s) used AND whether they are one- or two-sided<br>*Only common tests should be described solely by name; describe more complex techniques in the Methods section.*
☒	☐	A description of all covariates tested
☒	☐	A description of any assumptions or corrections, such as tests of normality and adjustment for multiple comparisons
☒	☐	A full description of the statistical parameters including central tendency (e.g. means) or other basic estimates (e.g. regression coefficient) AND variation (e.g. standard deviation) or associated estimates of uncertainty (e.g. confidence intervals)
☒	☐	For null hypothesis testing, the test statistic (e.g. *F*, *t*, *r*) with confidence intervals, effect sizes, degrees of freedom and *P* value noted<br>*Give P values as exact values whenever suitable.*
☒	☐	For Bayesian analysis, information on the choice of priors and Markov chain Monte Carlo settings
☒	☐	For hierarchical and complex designs, identification of the appropriate level for tests and full reporting of outcomes
☒	☐	Estimates of effect sizes (e.g. Cohen's *d*, Pearson's *r*), indicating how they were calculated

Our web collection on statistics for biologists contains articles on many of the points above.

Software and code

Policy information about availability of computer code

Data collection	Raw sequencing data (short-read and long-read) were processed using publicly available bioinformatics tools (bwa mem v0.7.17-r1188, samtools v1.13, SPAdes v3.13.1, MinION/Dorado (v1.0) and Guppy (v6.5.7), hifiasm (2025), Canu v2.2, Minimap2 v2.30, Racon v1.5.0, Pilon v1.24<br>All datasets analyzed were either newly generated in this study or obtained from publicly available repositories (GitHub, Zenodo).
Data analysis	Analyses were performed using standard open-source tools: RepeatMasker v4.1.2-p1, MAFFT v7.526, BLASTx, ORFfinder, DeviaTE, BEAST v2.7.5, bedtools v2.30.0, LTRharvest v1.6.5, Python3, VectorBuilder (2025), RStudio

For manuscripts utilizing custom algorithms or software that are central to the research but not yet described in published literature, software must be made available to editors and reviewers. We strongly encourage code deposition in a community repository (e.g. GitHub). See the Nature Portfolio guidelines for submitting code & software for further information.

Data

Policy information about availability of data

All manuscripts must include a data availability statement. This statement should provide the following information, where applicable:

- Accession codes, unique identifiers, or web links for publicly available datasets
- A description of any restrictions on data availability
- For clinical datasets or third party data, please ensure that the statement adheres to our policy

Data Availability:

Raw reads for D. melanogaster and D. erecta lines are available at NCBI under accession number PRJNA1359170, together with the D. erecta genome assembly. Raw data can be found in the associated Zenodo folder (https://zenodo.org/records/21804491). Publicly available data analysed in this study can be retrieved from NCBI at accession numbers PRJNA156883, PRJNA268111, PRJNA30085, PRJNA308584, PRJNA388788, PRJNA685394, PRJNA924845, PRJNA945389. Source data are provided with this paper.

Code Availability:
The analysis performed in this work have been documented with RMarkdown and have been made publicly available, together with the resulting figures and the library of D. erecta TE families consensus sequences generated with EarlGrey, in the associated Zenodo folder (https://zenodo.org/records/21804491).

Research involving human participants, their data, or biological material

Policy information about studies with human participants or human data. See also policy information about sex, gender (identity/presentation), and sexual orientation and race, ethnicity and racism.

Reporting on sex and gender	*Use the terms sex (biological attribute) and gender (shaped by social and cultural circumstances) carefully in order to avoid confusing both terms. Indicate if findings apply to only one sex or gender; describe whether sex and gender were considered in study design; whether sex and/or gender was determined based on self-reporting or assigned and methods used. Provide in the source data disaggregated sex and gender data, where this information has been collected, and if consent has been obtained for sharing of individual-level data; provide overall numbers in this Reporting Summary. Please state if this information has not been collected. Report sex- and gender-based analyses where performed, justify reasons for lack of sex- and gender-based analysis.*
Reporting on race, ethnicity, or other socially relevant groupings	*Please specify the socially constructed or socially relevant categorization variable(s) used in your manuscript and explain why they were used. Please note that such variables should not be used as proxies for other socially constructed/relevant variables (for example, race or ethnicity should not be used as a proxy for socioeconomic status). Provide clear definitions of the relevant terms used, how they were provided (by the participants/respondents, the researchers, or third parties), and the method(s) used to classify people into the different categories (e.g. self-report, census or administrative data, social media data, etc.) Please provide details about how you controlled for confounding variables in your analyses.*
Population characteristics	*Describe the covariate-relevant population characteristics of the human research participants (e.g. age, genotypic information, past and current diagnosis and treatment categories). If you filled out the behavioural & social sciences study design questions and have nothing to add here, write "See above."*
Recruitment	*Describe how participants were recruited. Outline any potential self-selection bias or other biases that may be present and how these are likely to impact results.*
Ethics oversight	*Identify the organization(s) that approved the study protocol.*

Note that full information on the approval of the study protocol must also be provided in the manuscript.

Field-specific reporting

Please select the one below that is the best fit for your research. If you are not sure, read the appropriate sections before making your selection.

☐ Life sciences ☐ Behavioural & social sciences ☒ Ecological, evolutionary & environmental sciences

For a reference copy of the document with all sections, see nature.com/documents/nr-reporting-summary-flat.pdf

Ecological, evolutionary & environmental sciences study design

All studies must disclose on these points even when the disclosure is negative.

Study description	This study investigates the discovery, evolutionary origin, and global invasion dynamics of Kuruka, a newly invading endogenous retrovirus (ERV) in Drosophila melanogaster. Using short- and long-read genomic data from over 1,000 D. melanogaster genomes collected between 2010 and 2025, as well as comparative genomic analyses across >1,500 drosophilid and arthropod assemblies, we reconstruct the invasion history, horizontal transfer event, and host defense response (piRNAs) associated with Kuruka.

Research sample	The research included genomic datasets from publicly available D. melanogaster populations worldwide (Africa, Asia, Europe, Oceania, and the Americas) and newly sequenced isofemale lines collected in 2023–2025 in North Dakota and Washington D.C., USA. Additional genomic data from D. erecta and D. simulans were used for phylogenetic and control analyses.
Sampling strategy	Population sampling was designed to capture both temporal and geographic variation in Kuruka's spread. Publicly available D. melanogaster short-read datasets representing global populations were re-analyzed for TE content, ensuring inclusion of pre-invasion and post-invasion genomes. Newly collected D. melanogaster lines (ND1–3, DW7, DC1) provided confirmation of ongoing activity. D. erecta was chosen as the putative donor species based on biogeographic and phylogenetic evidence. No statistical power analysis was required for this genomic survey.
Data collection	Short-read data were obtained from previously published studies [Grenier et al., 2015; Chen et al., 2024; Nunez et al., 2025]. Long-read sequencing was conducted on pooled and individual isofemale lines using Oxford Nanopore platforms (MinION and PromethION). DNA extractions followed standardized protocols for high-molecular-weight DNA. All newly generated data are deposited in public repositories (see Data availability).
Timing and spatial scale	The study integrates data from D. melanogaster populations collected between 1815 and 2025 across five continents, spanning the emergence and global spread of Kuruka. Field collections for long-read sequencing were conducted in 2023 (North Dakota) and 2025 (Fargo, ND and Washington D.C., USA). Comparative genomic analyses included drosophilid genomes from all major biogeographic realms.
Data exclusions	No data were excluded from the analysis.
Reproducibility	All computational analyses were performed with publicly available software using documented parameters. Custom scripts are available on GitHub (see "Software and code"). Analyses were replicated independently for multiple datasets (short- and long-read) and verified across different species and populations, yielding consistent results.
Randomization	Randomization was not applicable, as this was an observational genomic study rather than an experimental manipulation. Sample inclusion followed data availability and quality criteria rather than random assignment.
Blinding	Blinding was not applicable to this study. The analyses were performed using genomic and transcriptomic datasets, and no experimental group allocation or subjective measurements were involved.

Did the study involve field work? ☐ Yes ☒ No

Reporting for specific materials, systems and methods

We require information from authors about some types of materials, experimental systems and methods used in many studies. Here, indicate whether each material, system or method listed is relevant to your study. If you are not sure if a list item applies to your research, read the appropriate section before selecting a response.

Materials & experimental systems

n/a	Involved in the study
☒	☐ Antibodies
☒	☐ Eukaryotic cell lines
☒	☐ Palaeontology and archaeology
☐	☒ Animals and other organisms
☒	☐ Clinical data
☒	☐ Dual use research of concern
☒	☐ Plants

Methods

n/a	Involved in the study
☒	☐ ChIP-seq
☒	☐ Flow cytometry
☒	☐ MRI-based neuroimaging

Animals and other research organisms

Policy information about studies involving animals; ARRIVE guidelines recommended for reporting animal research, and Sex and Gender in Research

Laboratory animals	Drosophila melanogaster iso-female lines ND1–ND4, DW7, and DC1 were used. ND1–ND3 were collected in North Dakota in 2023; DW7 and DC1 were collected in Fargo, North Dakota, and Washington, D.C., respectively, in 2025. Established laboratory strains and publicly available resources were also used, including D. melanogaster w^1118^, D. simulans SZ129, and D. erecta strain 14021-0224.01.
Wild animals	Wild D. melanogaster flies were collected to establish iso-female lines in North Dakota in 2023 and in Fargo, North Dakota, and Washington, D.C., in 2025.
Reporting on sex	Sex was not used as an experimental variable and sex-specific analyses were not performed. The study used iso-female D. melanogaster lines. For D. erecta genome sequencing and small-RNA analysis, ovaries were dissected from approximately 300 flies; these samples were therefore female. Sex was otherwise not reported for the analysed samples.

Field-collected samples	Field-collected D. melanogaster samples were used to establish iso-female lines. Samples collected in North Dakota in 2023 were used for pooled Oxford Nanopore MinION sequencing. Two lines collected in Fargo, North Dakota, and Washington, D.C., in 2025 were sequenced separately.
Ethics oversight	The biological material used in this study consisted of established laboratory strains, publicly available resources, and newly collected D. melanogaster specimens. The study did not involve experiments on humans or vertebrate animals and therefore did not require ethical approval.

Note that full information on the approval of the study protocol must also be provided in the manuscript.

Plants

Seed stocks	*Report on the source of all seed stocks or other plant material used. If applicable, state the seed stock centre and catalogue number. If plant specimens were collected from the field, describe the collection location, date and sampling procedures.*
Novel plant genotypes	*Describe the methods by which all novel plant genotypes were produced. This includes those generated by transgenic approaches, gene editing, chemical/radiation-based mutagenesis and hybridization. For transgenic lines, describe the transformation method, the number of independent lines analyzed and the generation upon which experiments were performed. For gene-edited lines, describe the editor used, the endogenous sequence targeted for editing, the targeting guide RNA sequence (if applicable) and how the editor was applied.*
Authentication	*Describe any authentication procedures for each seed stock used or novel genotype generated. Describe any experiments used to assess the effect of a mutation and, where applicable, how potential secondary effects (e.g. second site T-DNA insertions, mosiacism, off-target gene editing) were examined.*
