# Supplementary material for: The ongoing invasion of the endogenous retrovirus Kuruka in natural Drosophila melanogaster populations
Source: Nat Commun. 2026 Sep 16;17:10789. doi: 10.1038/s41467-026-77761-0 (PMC13669377; doi:10.1038/s41467-026-77761-0)
Supplement: Supplementary file 3 [file 41467_2026_77761_MOESM3_ESM.pdf]

nature portfolio

Peer Review File

The ongoing invasion of the endogenous retrovirus *Kuruka* in natural *Drosophila melanogaster* populations

Corresponding Author: Dr Riccardo Pianezza

This file contains all reviewer reports in order by version, followed by all author rebuttals in order by version.

Version 0:

Reviewer comments:

Reviewer #1

(Remarks to the Author)
This manuscript presents the discovery and characterization of a novel and ongoing invasion of the Drosophila melanogaster genome by a Gypsy-family LTR retrotransposon. The authors identify the element by de novo assembly of genomic reads that fail to map to a reference genome, and then confirm it using long read data. Based on sequence similarity, they argue that it may have recently invaded from Drosophila yakuba. This is an interesting story, which will certainly be read and cited by those working on the evolutionary biology of transposable elements. The work is done well, and the paper is well-presented; I enjoyed reading it.
However, I think there are some aspects of the work that are potentially a bit 'thin' for such a high impact journal. That is, unless I am misunderstanding some aspects of what has been done, I think some parts of the (public) data could be analysed more completely to get a better handle on the yakuba origin. Given data already exist, I think this would should be quite easy to do.
Annoyingly, there are no line numbers.

Major:
(1) It is claimed on the basis of the D. yakuba genome that kuruka is present in single copy there, and based on genome assembly (and PCR) that it is 'absent' from simulans. However, it seems that this was not tested more thoroughly using raw reads from other isolates of these species. The authors use deviaTE to infer copy-number in melanogaster. Why not use this software to examine copy-number in all of the publicly available short-read data for the other members of the melanogaster subgroup (in addition to the reference genome)
(2) Kuruka silencing in Dmel via the piRNA pathway: If this is hypothesised to be the case, why not sequence piRNAs from the isofemale line that has the flamenco insertion (as done for erecta). Or, at least examine all of the publicly available sRNA data available for melanogaster to check whether anyone else has already done this.
(3) Figures 1 and 3 claim to be Bayesian phylogenetic trees inferred with BEAST. This seems unlikely, as the tree presented is not obviously clock-based (The tree figure doesn't have a scale with units for me to check), and extracting a tree in mutation-lengths from BEAST is not standard (nor could I see why it would be done here). However, the methods lack any description of tree inference. What was the substitution model? And, if BEAST was used, what was the clock model (strict? UCLD?) and prior, and what was the tree-shape model (coalescent? Birth-death?) and prior?

Minor:
(4) The term 'Endogenous retrovirus' is used in the title and abstract, but not explained until well into the body of the text. Anyone who is not intimately familiar with the Drosophila literature could think that this is a retrovirus that has become endogenous, like HERVs in humans (and ERVs in other mammals). Only once they have read quite a bit will they discover that this term is used here to be exchangeable with some LTR retrotransposons. Despite the statement that "The acquisition of the envelope transformed these elements from intracellular replicating retrotransposons into infectious retroviruses", I do not think gypsy is widely considered to be an infectious agent (i.e. transmitted among individuals as its primary mode of transmission)? If it were, surely we would regularly find them in somatic DNA but not germline cells, as we do true retroviruses such as HIV, FIV, SFV etc etc? Alternatively, the authors could provide citations in which it has been claimed to transmit between individuals (the closest I know is https://genesdev.cshlp.org/content/8/17/2046.full.pdf ?)
(5) 'Using a novel pipeline to …' and 'A similar strategy had previously been used to identify DNA viruses …' – I feel this rather overstates the novelty. The approach taken here is entirely identical to the approach used to discover DNA viruses in genome-sequencing data in many papers, including the one by Wallace et al. The only difference is a blast search against

TE sequences in place of a blast search against viral sequences. I'm not sure that counts as a 'novel strategy'.
(6) End of first paragraph of section 2.5 – I think the intended word is 'contiguity'
(7) "If D. erecta is the donor of the HT, our results would imply that a silenced TE, with just a single copy in a genome, may still be horizontally transmitted and trigger a novel invasion in a naive host. This is not impossible …" To be transmitted from this insertion, the TE would need to be expressed on its own and in its sense orientation (it would need an RT and integrase), but isn't expression from this locus as a 'unistrand' controlled by the host? Could one not look at RNAseq from Dyak to decide if this is credible?

Reviewer #2

(Remarks to the Author)
Review of
"The ongoing invasion of the endogenous retrovirus Kuruka in natural Drosophila melanogaster populations" by Kofler and colleagues

Transposable elements are typically inherited vertically through the germline genome; however, on rare occasions they can also move horizontally between species. The vectors and mechanisms that enable such horizontal transfer events, thereby initiating a de novo transposon invasion in a new host species, remain poorly understood. Recent work, primarily from the Kofler laboratory, has demonstrated in several publications that horizontal transfer of transposable elements in Drosophila melanogaster has occurred surprisingly frequently, at least over the last ~200 years.

In the present manuscript, the authors report what they describe as the most recent invasion of a transposable element into D. melanogaster: a retrotransposon termed Kuruka.

The topic of horizontal transfer of transposable elements is timely and of broad interest, and the manuscript convincingly demonstrates that Kuruka is currently invading D. melanogaster populations on a global scale. The central claims are largely supported by the oresented data, most of which are computational in nature.

Novelty and impact

This manuscript represents the latest in a series of publications from the Kofler lab reporting horizontal transfer events from other Drosophila species into D. melanogaster. In this context, the conceptual novelty is limited.

What distinguishes the present work from earlier reports is that the Kuruka invasion appears to be ongoing, that silencing has not yet been fully established in all populations, and the claim that this represents the first documented invasion into the now cosmopolitan D. melanogaster species originating from within Africa (with D. erecta proposed as the source species), which is the ancestral range of melanogaster. These are interesting observations; however, the study does not substantially advance our mechanistic understanding of horizontal transfer itself, nor does it present new insights beyond the current literature into how the host responds to such an invasion.

Major claims

Identification of newly invading TEs from short-read data
The manuscript convincingly demonstrates that short-read genome sequencing data can be used to identify newly invading transposable elements. This represents a useful addition to the computational toolkit for TE discovery in population genomic datasets. That said, the novelty of this approach is somewhat reduced by the fact that conceptually similar strategies have been employed previously, as also cited by the authors.

Reconstruction of the Kuruka invasion
The reconstruction of the invasion timeline and geographic spread of Kuruka is well executed. One caveat concerns the inferred African origin of the invasion, which—if I understand correctly—is supported by a single sampled population. As the broader claim that this is the first invasion of a TE into D. melanogaster within Africa hinges critically on this point, the robustness of this interpretation may warrant a more cautious framing.

Origin of Kuruka in D. erecta
Based on the data presented, D. erecta appears to be the most plausible source species. However, the D. erecta genome harbors only a single Kuruka insertion, which is located within the flamenco piRNA cluster. It is unclear whether this insertion is structurally intact, and its location suggests that Kuruka is likely silenced in this species. This makes a horizontal transfer scenario from D. erecta somewhat less straightforward. The authors appropriately acknowledge this limitation, but it nevertheless weakens the strength of the inference regarding the origin species.

Silencing of Kuruka via flamenco insertions
The proposed model that Kuruka is silenced through insertions into the flamenco piRNA cluster is plausible, but currently rests primarily on presence/absence calls of Kuruka insertions within flamenco. It remains unclear whether populations lacking flamenco insertions nonetheless exhibit Kuruka silencing (for example, one of the North Dakota samples appears to contain Kuruka insertions but none in flamenco). More generally, the novelty of this claim is diminished by recent work from the authors on Shellder, as well as by the Tirant invasion study by Senti and colleagues, both of which report that flamenco insertions are associated with somatic silencing and that strong silencing can also arise from non-flamenco insertions.

Other comments

Figure 1A: Have the authors attempted to infer the env splice site by alignment to related elements? Explicitly noting that env is spliced in errantiviruses would be helpful.

Figure 1C: Why were only a subset of D. melanogaster retrotransposons included in this phylogenetic analysis, and what was the rationale for their selection? Given the limited sampling, the inferred relationship to Tirant is not entirely clear.

Are the Kuruka insertions in flamenco consistently antisense relative to the direction of transcription?

The relevant control strain is w^1118, not w^118 (throughout the manuscript).

Small RNA data: The small RNA-seq profiles appear unusually "peaky," including for TE-derived piRNAs. It would strengthen this section to include analyses of ping-pong signatures and phasing, ideally using statistical z-scores to assess significance.

How was the flamenco piRNA cluster annotated in D. erecta? Showing genome-uniquely mapping piRNAs alongside the annotation would be informative.

Figures: Figure legends and labeling, particularly for the supplementary figures, are often quite minimal and could be expanded to improve clarity and self-containment.

Version 1:

Reviewer comments:

Reviewer #1

(Remarks to the Author)
The reviewers have suitably addressed the majority of my points, and I have no further major comments.

I still believe the presentation of the BEAST analysis is inappropriate, but I'm not going to get into an argument about it - I'm happy for the readership to judge. [Under a strict clock with no rate information BEAST simply sets the rate to 1 sub/site/unit_time. The branch lengths may well then be the *expected* number of substitutions per site on that branch *under a strict clock*. But that is wholly different from the number of substitutions per site (as currently labelled) that have happened, or that you would get for a non-clock Bayesian or ML analysis. It is therefore odd to label it as the "number of substitutions". The methods and legend phrasing seems to confound 'Bayesian' with 'Clock-based', and focus on the former (which is basically irrelevant) and ignore the latter (which is dominating the appearance of the tree). More seriously, if a clock has been fitted then it has a root, but the trees are presented unrooted. If they want to force a clock, they should present it as such. As things stand, this is simply making the (probably very noisy) tree look MUCH prettier than it should.

Minor point: the majority of the fonts in the majority of the figures are too small.

Reviewer #2

(Remarks to the Author)
The authors did a careful job in addressing the reviewer's comments.
Panel 3b might benefit from some more labelling (marker size, expected size of PCR product, etc.)
Overall, I consider the manuscript ready for publication.
congrats on an interesting story.

Open Access This Peer Review File is licensed under a Creative Commons Attribution 4.0 International License, which permits use, sharing, adaptation, distribution and reproduction in any medium or format, as long as you give appropriate credit to the original author(s) and the source, provide a link to the Creative Commons license, and indicate if changes were made.
In cases where reviewers are anonymous, credit should be given to 'Anonymous Referee' and the source.
The images or other third party material in this Peer Review File are included in the article's Creative Commons license, unless indicated otherwise in a credit line to the material. If material is not included in the article's Creative Commons license and your intended use is not permitted by statutory regulation or exceeds the permitted use, you will need to obtain permission directly from the copyright holder.
To view a copy of this license, visit https://creativecommons.org/licenses/by/4.0/

REVIEWER COMMENTS

Reviewer #1 (Remarks to the Author):

This manuscript presents the discovery and characterization of a novel and ongoing invasion of the Drosophila melanogaster genome by a Gypsy-family LTR retrotransposon. The authors identify the element by de novo assembly of genomic reads that fail to map to a reference genome, and then confirm it using long read data. Based on sequence similarity, they argue that it may have recently invaded from Drosophila yakuba. This is an interesting story, which will certainly be read and cited by those working on the evolutionary biology of transposable elements. The work is done well, and the paper is well-presented; I enjoyed reading it.

However, I think there are some aspects of the work that are potentially a bit 'thin' for such a high impact journal. That is, unless I am misunderstanding some aspects of what has been done, I think some parts of the (public) data could be analysed more completely to get a better handle on the yakuba origin. Given data already exist, I think this would should be quite easy to do.

Annoyingly, there are no line numbers.

We apologize for the missing line numbers, in the revised version they are present.

Major:

(1) It is claimed on the basis of the D. yakuba genome that kuruka is present in single copy there, and based on genome assembly (and PCR) that it is 'absent' from simulans. However, it seems that this was not tested more thoroughly using raw reads from other isolates of these species. The authors use deviaTE to infer copy-number in melanogaster. Why not use this software to examine copy-number in all of the publicly available short-read data for the other members of the melanogaster subgroup (in addition to the reference genome)

We followed the reviewer's suggestion, and downloaded a total of 62 whole-genome sequencing short-reads samples from species of the simulans clade (29 *D. simulans*, 21 *D. sechellia* and 12 *D. maurititana*) to confirm the absence of *Kuruka* in these species. *Kuruka* is indeed absent from all of the tested samples, with copynumbers estimated consistently below 1. The few reads mapping to the Kuruka consensus in these species are rich of SNPs compared to the consensus, suggesting that only a few degraded fragments might be present in those genomes, similarly to the *D. melanogaster* samples collected before the new *Kuruka* invasion.

We included a new Supplementary Figure (now Figure S7) to show these results, and updated the results and M&M sections accordingly.

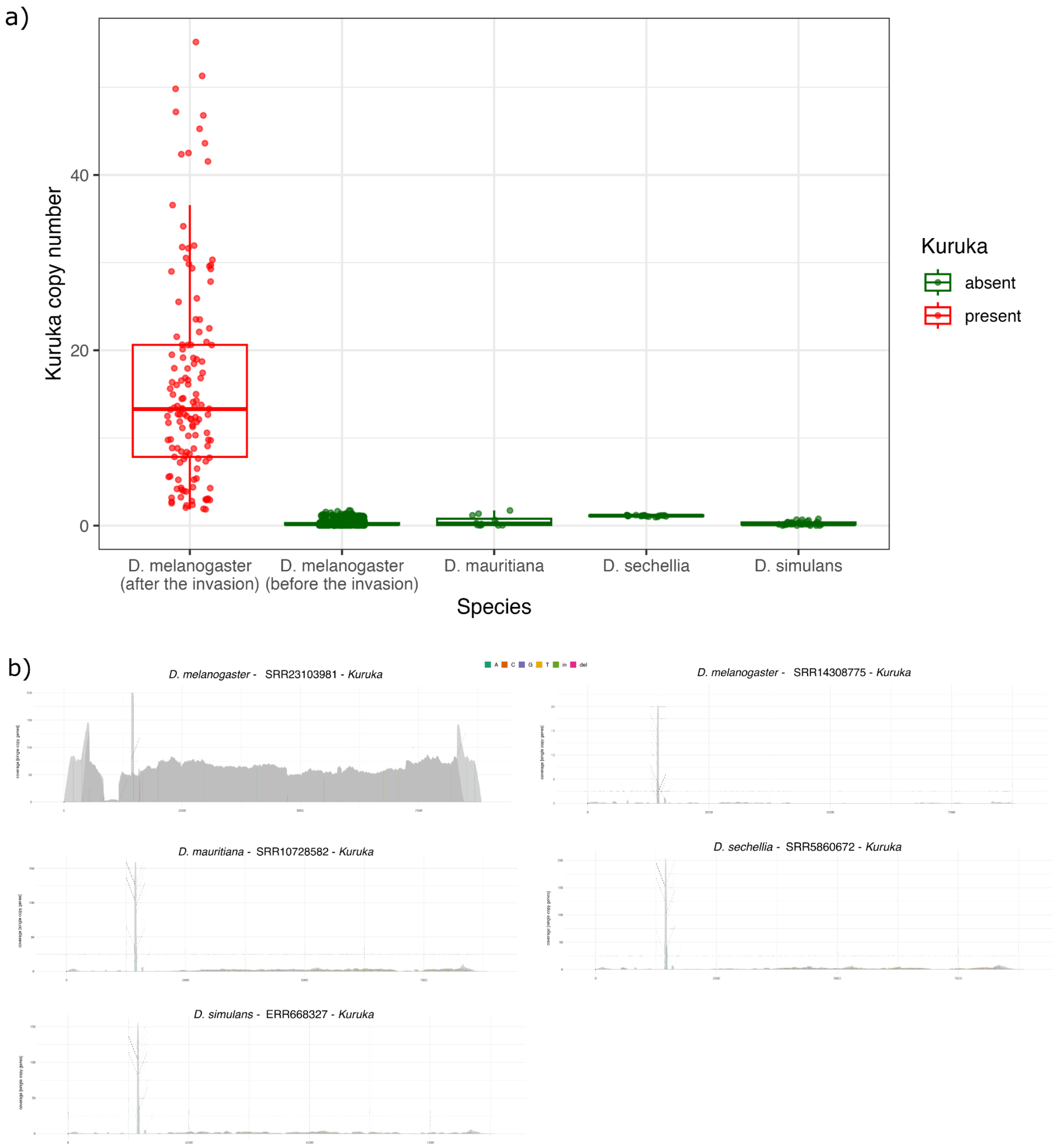

(2) Kuruka silencing in Dmel via the piRNA pathway: If this is hypothesised to be the case, why not sequence piRNAs from the isofemale line that has the flamenco insertion (as done for erecta). Or, at least examine all of the publicly available sRNA data available for melanogaster to check whether anyone else has already done this.

We followed the reviewers suggestion and sequenced the small RNAs of two strains sampled 2025 in the USA (DC1 and DW7). Both strains have *Kuruka* piRNA whereas strains sampled before the invasion (I17 Ithaca 2004 and B10 Beijing 2010) do not have *Kuruka* piRNAs. This is shown in a novel supplementary figure.

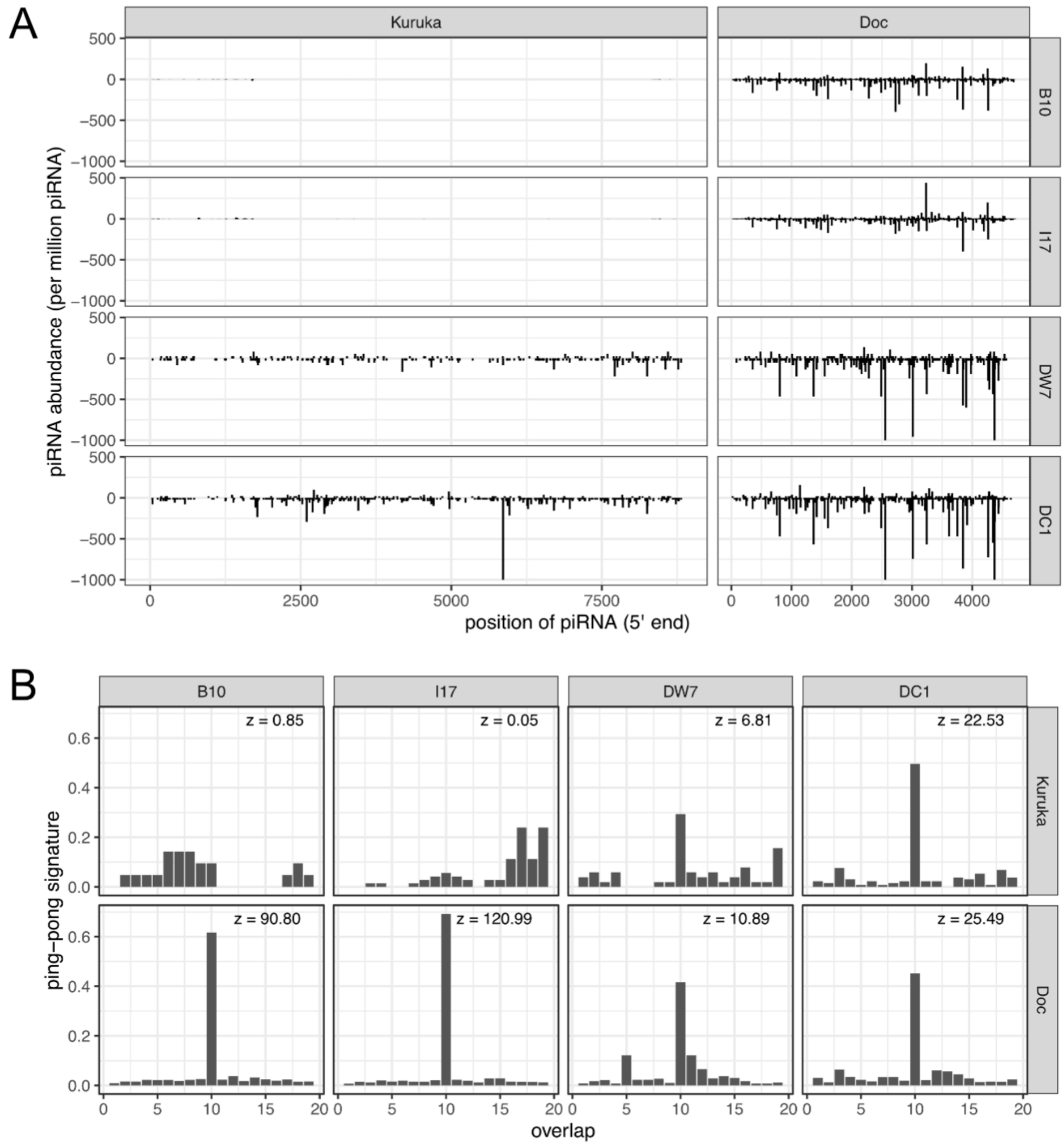

Figure 5: Abundance of piRNAs for *Kuruka* and *Doc* in *D. melanogaster* strains collected at different years and geographic regions (I17 Ithaca, USA 2004; B10 Bejing, China 1995; DC1 Washington DC, USA, 2025; DW7 North Dakota, USA, 2025). A) Distribution of piRNAs along the TE sequence. Sense piRNAs are shown on the positive y-axis and antisense piRNAs on the negative y-axis B) *Kuruka* shows a ping-pong signature in DW7 and DC1. Ping-pong signatures for somatic TEs, such as the related *Tirant*, were observed before [Rafanel et al., 2026].

(3) Figures 1 and 3 claim to be Bayesian phylogenetic trees inferred with BEAST. This seems unlikely, as the tree presented is not obviously clock-based (The tree figure doesn't have a scale with units for me to check), and extracting a tree in mutation-lengths from BEAST is not standard (nor could I see why it would be done here). However, the methods lack any description of tree inference. What was the substitution model? And, if BEAST was used, what was the clock model (strict? UCLD?) and prior, and what was the tree-shape model (coalescent? Birth-death?) and prior?

Phylogenetic trees were inferred in a Bayesian framework using BEAST. For nucleotide data (Figure 3), sequence evolution was modeled using the JC69 substitution model, while

protein sequences (Figure 1) were analyzed under the LG amino-acid substitution model. A strict molecular clock with the rate fixed to 1 was used in all analyses, and tree topology was inferred under a Yule (pure-birth) tree prior. As no temporal calibration was applied, branch lengths represent expected substitutions per site rather than time. To avoid ambiguity, we have added a scale bar to the figures and expanded the Methods section to explicitly describe the models used (lines 378-382).

Minor:

(4) The term 'Endogenous retrovirus' is used in the title and abstract, but not explained until well into the body of the text. Anyone who is not intimately familiar with the Drosophila literature could think that this is a retrovirus that has become endogenous, like HERVs in humans (and ERVs in other mammals). Only once they have read quite a bit will they discover that this term is used here to be exchangeable with some LTR retrotransposons. Despite the statement that "The acquisition of the envelope transformed these elements from intracellular replicating retrotransposons into infectious retroviruses", I do not think gypsy is widely considered to be an infectious agent (i.e. transmitted among individuals as its primary mode of transmission)? If it were, surely we would regularly find them in somatic DNA but not germline cells, as we do true retroviruses such as HIV, FIV, SFV etc etc? Alternatively, the authors could provide citations in which it has been claimed to transmit between individuals (the closest I know is https://genesdev.cshlp.org/content/8/17/2046.full.pdf ?)

We reorganized the introduction to explain in the first paragraph what we exactly refer to when we talk about "endogenous retroviruses" in *Drosophila* (now lines 28-33).

To avoid confusion, we removed the statement about "infectivity", as we explain anyway what we mean later, when we discuss the iERVs ability to move from somatic to germline tissues.

(5) 'Using a novel pipeline to …' and 'A similar strategy had previously been used to identify DNA viruses …' – I feel this rather overstates the novelty. The approach taken here is entirely identical to the approach used to discover DNA viruses in genome-sequencing data in many papers, including the one by Wallace et al. The only difference is a blast search against TE sequences in place of a blast search against viral sequences. I'm not sure that counts as a 'novel strategy'.

We agree with the reviewer that the underlying strategy is not methodologically novel. Rather than introducing a new computational strategy, our contribution lies in applying this existing pipeline to the detection of TE invasions, a context in which it has not previously been used.

To avoid overstating novelty, we have revised the opening sentence of the Results (line 88):

"To test whether novel TE invasions were captured by short-read data from recently sampled *D. melanogaster* strains, **we adapted a strategy** originally designed to identify DNA viruses in *Drosophila* sequencing data (Wallace2021)."

(6) End of first paragraph of section 2.5 – I think the intended word is 'contiguity'

We fixed the mistake.

(7) "If D. erecta is the donor of the HT, our results would imply that a silenced TE, with just a single copy in a genome, may still be horizontally transmitted and trigger a novel invasion in a naive host. This is not impossible …" To be transmitted from this insertion, the TE would need to be expressed on its own and in its sense orientation (it would need an RT and integrase), but isn't expression from this locus as a 'unistrand' controlled by the host? Could one not look at RNAseq from Dyak to decide if this is credible?

HT likely involves two steps: first, the transmission of the TE from the donor-species to the vector mediating the HT (e.g. viruses), followed by a second transfer from the vector to the novel host species. For the current discussion, i.e. whether a TE silenced by the donor species could be subject to HT, largely the first step is important. It is indeed possible that silencing by the piRNA based host defense diminishes the chances of a TE to get transmitted to potential vectors. However, it does not preclude transmission, since it is feasible that the transmission to the vector happens in non-gonadal somatic tissues where the piRNA based host defense is typically not active. Additionally, it is also possible that the transmission to vectors happens by a mechanism that does not require transcription of the TE, like microhomology-mediated recombination (Gilbert 2016).

To make this more clear we added the following to the discussion (lines 275-282):

"This is not impossible, as activity of the TE in the gonads of the donor species may not be necessary for HT. The transfer of *Kuruka* into vectors for HT may happen in any non-gonadal somatic cell, where the piRNA pathway is typically not active (Fagegaltier2016, Jones2016), or alternatively it may be mediated by transcription-independent mechanisms such as microhomology-mediated recombination, which for example led to integrations of host TEs into baculoviruses (i.e. strong candidates for vectors that may mediate HT (Gilbert2016)."

Reviewer #2 (Remarks to the Author):

Review of

"The ongoing invasion of the endogenous retrovirus Kuruka in natural Drosophila melanogaster populations" by Kofler and colleagues

Transposable elements are typically inherited vertically through the germline genome; however, on rare occasions they can also move horizontally between species. The vectors and mechanisms that enable such horizontal transfer events, thereby initiating a de novo transposon invasion in a new host species, remain poorly understood. Recent work, primarily from the Kofler laboratory, has demonstrated in several publications that horizontal

transfer of transposable elements in Drosophila melanogaster has occurred surprisingly frequently, at least over the last ~200 years.

In the present manuscript, the authors report what they describe as the most recent invasion of a transposable element into D. melanogaster: a retrotransposon termed Kuruka.

The topic of horizontal transfer of transposable elements is timely and of broad interest, and the manuscript convincingly demonstrates that Kuruka is currently invading D. melanogaster populations on a global scale. The central claims are largely supported by the oresented data, most of which are computational in nature.

Novelty and impact

This manuscript represents the latest in a series of publications from the Kofler lab reporting horizontal transfer events from other Drosophila species into D. melanogaster. In this context, the conceptual novelty is limited.

What distinguishes the present work from earlier reports is that the Kuruka invasion appears to be ongoing, that silencing has not yet been fully established in all populations, and the claim that this represents the first documented invasion into the now cosmopolitan D. melanogaster species originating from within Africa (with D. erecta proposed as the source species), which is the ancestral range of melanogaster. These are interesting observations; however, the study does not substantially advance our mechanistic understanding of horizontal transfer itself, nor does it present new insights beyond the current literature into how the host responds to such an invasion.

We thank the reviewer for this thoughtful assessment. We agree that our study does not uncover the molecular mechanism underlying horizontal transfer itself, nor does it identify an entirely new host defense pathway. Rather, we view the main advances of our work as several conceptual breakthroughs.

- First, our study documents, to our knowledge, **the first ongoing TE invasion in a natural population of any organism** (with the possible exception of *KoRV* in koalas, although it remains unclear whether *KoRV* is currently spreading through populations). We show that the endogenous retrovirus *Kuruka* is presently spreading in global *D. melanogaster* populations. Using genomic time-series data, which are not available for koalas, we demonstrate that *Kuruka* was absent only a few years ago and is now gradually spreading through global populations where it has not yet fully penetrated all populations. Such systems are exceptionally rare because most TE invasions are reconstructed retrospectively, after the invasion has already been completed. *Kuruka* therefore provides a unique opportunity to study the earliest

phases of TE-host interactions in real time and in natural populations. This ongoing invasion will also offer a unique opportunity for the research community to investigate the establishment of host defense mechanisms in natural populations.

- Second, we reconstruct the *Kuruka* invasion at high spatiotemporal resolution, documenting its emergence in sub-Saharan Africa and subsequent spread to other continents. Our study therefore provides **the first detailed reconstruction of the spatiotemporal spread of an endogenous retrovirus**.

- Third, our data indicate that *Kuruka* most likely originated from an Afrotropical donor species, with *D. erecta* being the most plausible source. In contrast to previous invasions, which originated from species endemic to South America or from the cosmopolitan *D. simulans*, we therefore describe **the first documented invasion involving an African donor species**. This finding is important because Africa is the ancestral range of *D. melanogaster*. Previous work has speculated that habitat expansions may be an important force driving recent invasions. Our finding that a species from the ancestral range can act as a donor of horizontal transfer suggests that habitat expansions are not necessary for triggering invasions, thereby providing important clues about the proximal causes and factors responsible for horizontal transfer.

- Finally, the finding that the putative donor species harbors only a single, silenced copy of *Kuruka* in a somatic piRNA cluster provides the first example of the resurrection of a "dead" transposable element. This observation highlights that **"dead" TEs may be revived by horizontal transfer**, allowing them to escape host silencing and multiply in naïve hosts that have not yet established effective silencing mechanisms. TEs may therefore be much more resilient over evolutionary timescales than previously thought.

Major claims

Identification of newly invading TEs from short-read data

The manuscript convincingly demonstrates that short-read genome sequencing data can be used to identify newly invading transposable elements. This represents a useful addition to the computational toolkit for TE discovery in population genomic datasets. That said, the novelty of this approach is somewhat reduced by the fact that conceptually similar strategies have been employed previously, as also cited by the authors.

Reconstruction of the Kuruka invasion

The reconstruction of the invasion timeline and geographic spread of Kuruka is well executed. One caveat concerns the inferred African origin of the invasion, which—if I understand correctly—is supported by a single sampled population. As the broader claim that this is the first invasion of a TE into D. melanogaster within Africa hinges critically on this point, the robustness of this interpretation may warrant a more cautious framing.

We thank the reviewer for raising this point. The inference of an African origin is supported by two independent lines of evidence.

First, Kuruka-like insertions are detected exclusively in Afrotropical *Drosophila* species and are absent from all non-African species surveyed (Figure 3A), strongly suggesting an African source. Second, within *D. melanogaster*, Kuruka insertions are present in five independently sampled African populations prior to their appearance elsewhere (Figure 2B-C), so the signal is not driven by a single population. Together, these observations make an African origin of the invasion the most parsimonious interpretation.

We made it more clear in the text:

"Kuruka first appeared in **some populations sampled from** Africa in 2010" (line 141)

"Interestingly, all species having insertions strongly resembling *Kuruka* are Afrotropical *Drosophila* species of the melanogaster group**, further supporting its African origin.**" (line 170)

Origin of Kuruka in D. erecta

Based on the data presented, D. erecta appears to be the most plausible source species. However, the D. erecta genome harbors only a single Kuruka insertion, which is located within the flamenco piRNA cluster. It is unclear whether this insertion is structurally intact, and its location suggests that Kuruka is likely silenced in this species. This makes a horizontal transfer scenario from D. erecta somewhat less straightforward. The authors appropriately acknowledge this limitation, but it nevertheless weakens the strength of the inference regarding the origin species.

We tested the structural integrity and found that *Kuruka* in *D. erecta* encodes the same ORFs and protein as in *D. melanogaster*.

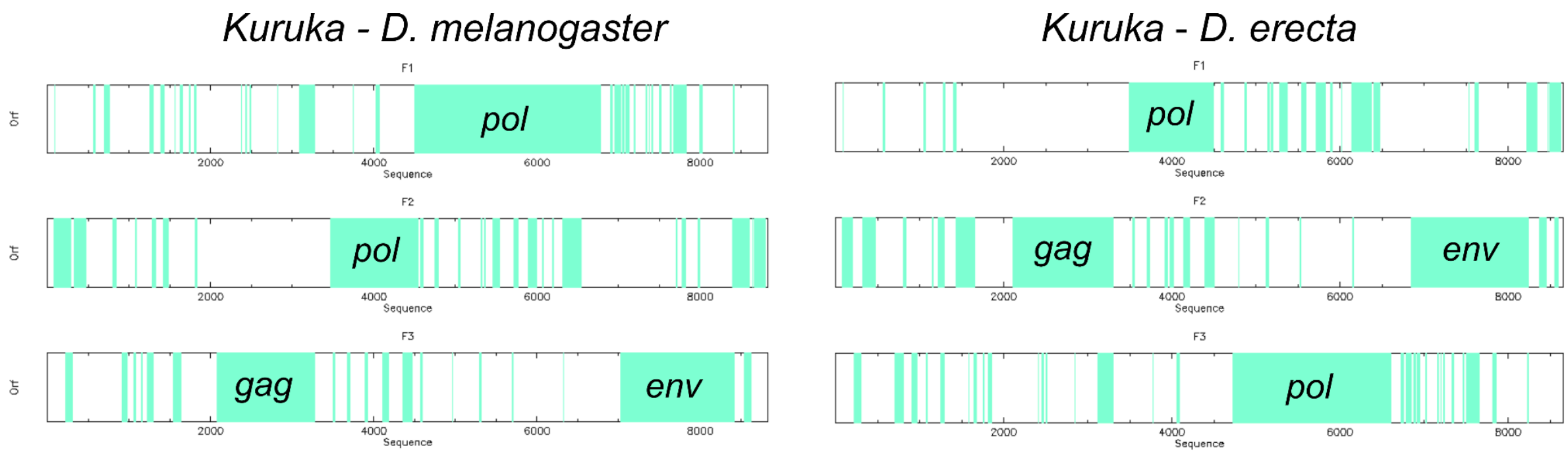

We added the novel results to the supplement and referred to it in the text (line 206-207).

Additionally, we added the following explanation to the discussion which highlights that silencing of a TE in the host gonads (as *Kuruka* by flamenco in *D. erecta*) may not make HT impossible (see also comment to reviewer 1):

“This is not impossible, as activity of the TE in the gonads of the donor species may not be necessary for HT. The transfer of *Kuruka* into vectors for HT may happen in any non-gonadal somatic cell, where the piRNA pathway is typically not active (Fagegaltier2016, Jones2016), or alternatively it may be mediated by transcription-independent mechanisms such as microhomology-mediated recombination, which for example led to integrations of host TEs into baculoviruses (i.e. strong candidates for vectors that may mediate HT (Gilbert2016).” (lines 275-282)

Silencing of Kuruka via flamenco insertions

The proposed model that Kuruka is silenced through insertions into the flamenco piRNA cluster is plausible, but currently rests primarily on presence/absence calls of Kuruka insertions within flamenco. It remains unclear whether populations lacking flamenco insertions nonetheless exhibit Kuruka silencing (for example, one of the North Dakota samples appears to contain Kuruka insertions but none in flamenco). More generally, the novelty of this claim is diminished by recent work from the authors on Shellder, as well as by the Tirant invasion study by Senti and colleagues, both of which report that flamenco insertions are associated with somatic silencing and that strong silencing can also arise from non-flamenco insertions.

The reviewer is correct. To address this we sequenced the small RNAs of two strains sampled 2025 in the USA (DC1 and DW7). Both strains have Kuruka piRNAs, whereas strains sampled before the invasion (I17 Ithaca 2004 and B10 Beijing 2010) do not have Kuruka piRNAs. Indeed, as speculated by the reviewer, the strain without flamenco insertion (DW7) does also generate Kuruka piRNA, in agreement with the findings of Senti and colleagues. *Kuruka* further has a ping-pong signature in DC1 and DW7, again in agreement with the findings of Senti and colleagues, where the closely related somatic transposon, *Tirant*, also has a ping-pong signature (https://link.springer.com/article/10.1038/s44318-026-00777-1).

This result is shown in a novel supplementary figure and referred to in the text (lines 153-154).

“However, both strains generated piRNA for Kuruka (Fig. S5). The production of piRNAs in the strain without flamenco insertion could be due to insertions in the 3'-UTR (Rafanel2025).”

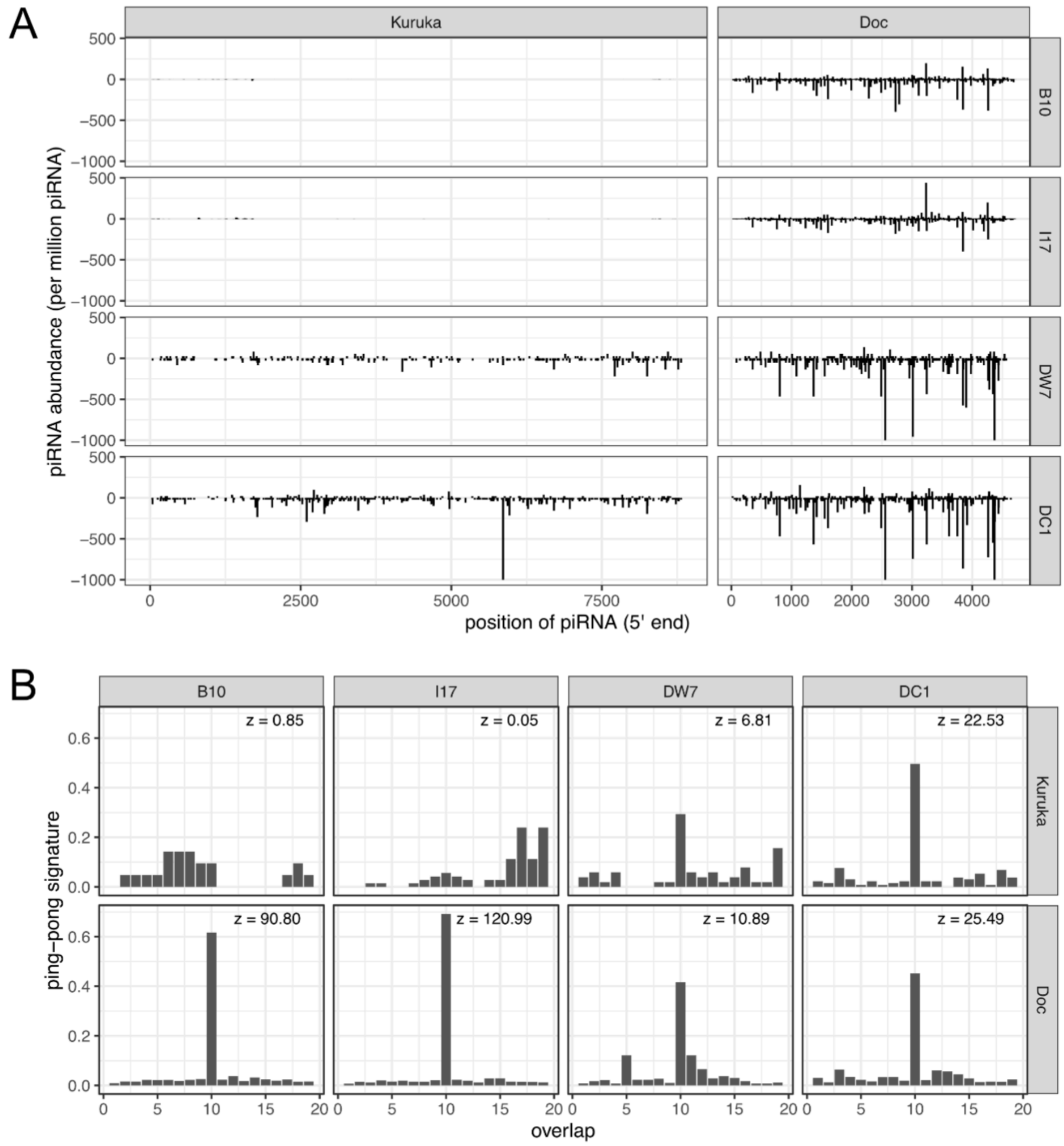

Figure 5: Abundance of piRNAs for *Kuruka* and *Doc* in *D. melanogaster* strains collected at different years and geographic regions (I17 Ithaca, USA 2004; B10 Bejing, China 1995; DC1 Washington DC, USA, 2025; DW7 North Dakota, USA, 2025). A) Distribution of piRNAs along the TE sequence. Sense piRNAs are shown on the positive y-axis and antisense piRNAs on the negative y-axis B) *Kuruka* shows a ping-pong signature in DW7 and DC1. Ping-pong signatures for somatic TEs, such as the related *Tirant*, were observed before [Rafanel et al., 2026].

Other comments

Figure 1A: Have the authors attempted to infer the env splice site by alignment to related elements? Explicitly noting that env is spliced in errantiviruses would be helpful.

We added this information in the introduction (lines 32-33): "Within this superfamily, some TEs contain an envelope gene, thought to be derived from DNA Baculoviruses (Terzian2001,

Rohrmann2001), **and like their baculoviral relatives, express this envelope protein from a spliced subgenomic mRNA (**Pelisson1994)”

To find conserved splicing sites, we aligned the sequence of *Kuruka* to those of related elements (*Tirant* and *Gypsy5*). We added the sequence of *Gypsy*, where the splicing sites were directly annotated and could be used as reference (Pelisson1994). The alignment below highlights the region corresponding to the annotated splicing donor and acceptor sites in *Gypsy*.

While no obvious conserved donor site emerges, the acceptor site is extremely well conserved in all of the elements, including *Kuruka*.

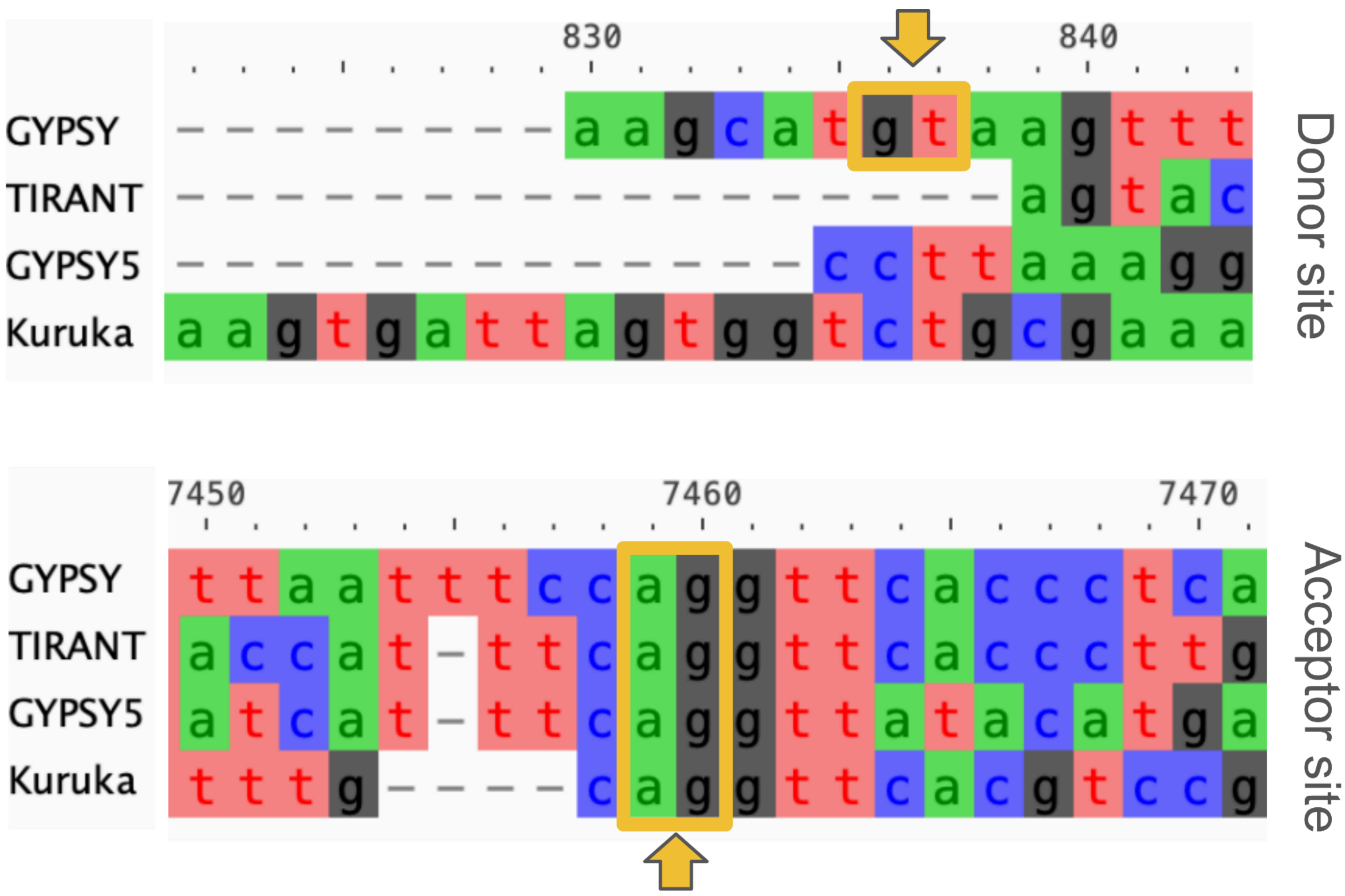

We added this figure in the supplement and updated the results section (lines 121-125):

“To test whether the *env* splicing observed for some errantiviruses such as Gypsy (*Pelisson1994*) is conserved in *Kuruka* we aligned the sequence several related errantiviruses (*Kuruka*, *Gypsy*, *Tirant* and *Gypsy5*). While no obvious conserved splice donor site is apparent, the splice acceptor site is extremely well conserved across all elements, including *Kuruka*.”

Figure 1C: Why were only a subset of D. melanogaster retrotransposons included in this phylogenetic analysis, and what was the rationale for their selection? Given the limited sampling, the inferred relationship to Tirant is not entirely clear.

We initially included only a subset of *D. melanogaster* retrotransposons because our primary goal was not to resolve the detailed phylogenetic relationships among drosophilid transposable elements, but rather to assign *Kuruka* to its appropriate superfamily. However, as we subsequently stated that *Kuruka* clusters closely with *Tirant*, we agree with the reviewer that this claim requires a more comprehensive and careful phylogenetic analysis.

Based on its genomic features —most notably the presence of an envelope gene— and on sequence similarity searches, *Kuruka* can be unambiguously assigned to the *Gypsy*/*Gypsy* clade. The only TEs showing detectable sequence similarity to *Kuruka* are *Tirant* and *Gypsy5*, which also belong to this clade. For this reason, rather than expanding the analysis to include TEs from other, more distantly related clades, we revised the phylogenetic analysis to include all currently known *Gypsy*/*Gypsy* elements in *D. melanogaster.*

The resulting tree showed that the most closely related TE is actually *Gypsy5*, refining the phylogenetic relationships of Kuruka within the *Gypsy*/*Gypsy* clade. We updated Figure 1 with the new tree and the text with the new result.

c)

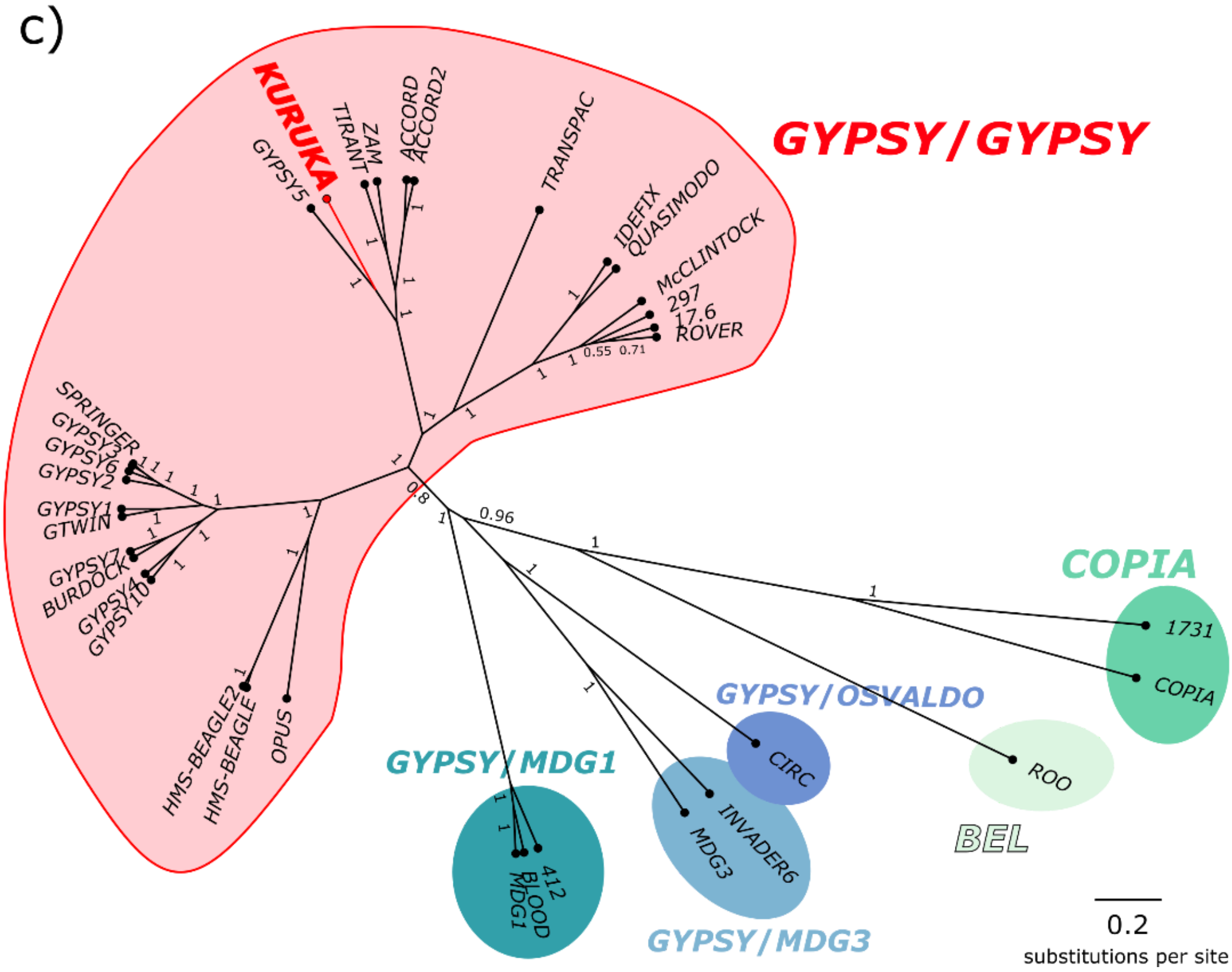

Are the Kuruka insertions in flamenco consistently antisense relative to the direction of transcription?

Yes, the insertions of *Kuruka* in *flamenco* are antisense both in *D. melanogaster* and *D. erecta.* We highlighted the orientation of these insertions with arrows both in the main manuscript (Fig. 4) and in the Supplementary figures.

The relevant control strain is w^1118, not w^118 (throughout the manuscript).

We thank the reviewer for the careful correction. We fixed the strain name throughout the manuscript.

Small RNA data: The small RNA-seq profiles appear unusually "peaky," including for TE-derived piRNAs. It would strengthen this section to include analyses of ping-pong signatures and phasing, ideally using statistical z-scores to assess significance.

We are now showing z-scores for all ping-pong signatures, including the ping-pong signatures in the novel supplementary figure 5. We also added ping-pong signatures and z-scores to supplementary figures 11 and 12.

We are not showing the phasing signatures as we do not think it is adding any important messages to the manuscript (see for example the plot below, which shows the phasing signature of *Kuruka* and *Doc* in four different *D. melanogaster* strains).

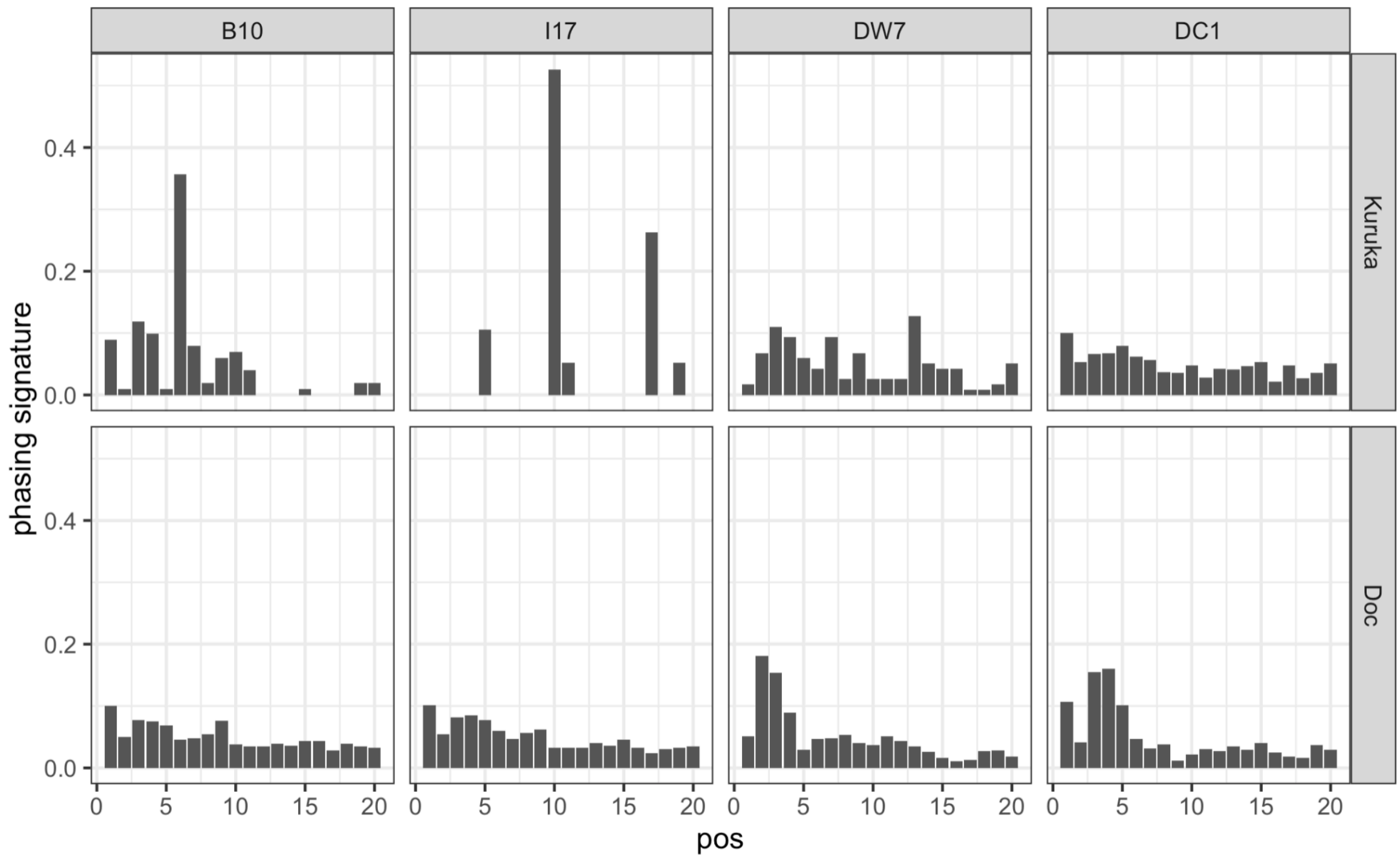

How was the flamenco piRNA cluster annotated in D. erecta? Showing genome-uniquely mapping piRNAs alongside the annotation would be informative.

To annotate *flamenco* in the *D. erecta*, we relied on the coordinates of *flamenco* in the *D. erecta* reference genome found by Lopik et al. We retrieved the sequence of *flamenco* and mapped it to our assembly.

Following the reviewers' suggestion, to corroborate the robustness of the annotation we mapped piRNAs from *D. erecta* to our assembly. Coverage of the uniquely mapped piRNAs

is shown in the figure below, highlighting a hotspot of piRNAs corresponding to the *flamenco* annotation.

Interestingly, the right part of *flamenco* seems to be producing piRNAs also in the embryo sample. The *Kuruka* insertion lies in the left part of the cluster, purely somatically expressed as it is absent in the embryo piRNAs. The insertion is antisense (see black arrow).

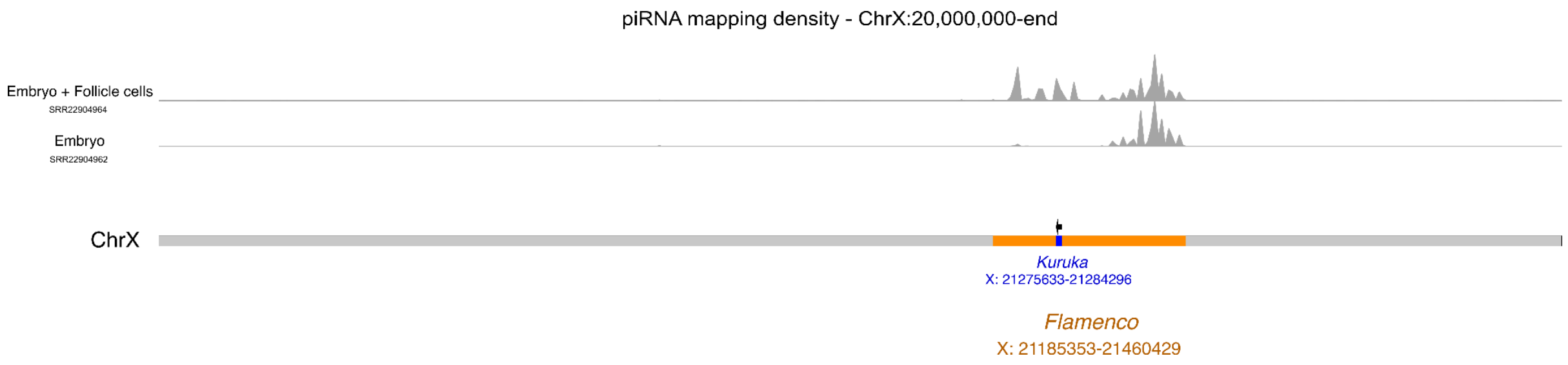

We added the novel figure to the supplement.

Figures: Figure legends and labeling, particularly for the supplementary figures, are often quite minimal and could be expanded to improve clarity and self-containment.

We have revised and expanded all figure legends, particularly those of the supplementary figures, to improve clarity and make them more fully self-contained.

REVIEWER COMMENTS

Reviewer #1 (Remarks to the Author):

The reviewers have suitably addressed the majority of my points, and I have no further major comments.

I still believe the presentation of the BEAST analysis is inappropriate, but I'm not going to get into an argument about it - I'm happy for the readership to judge. [Under a strict clock with no rate information BEAST simply sets the rate to 1 sub/site/unit_time. The branch lengths may well then be the *expected* number of substitutions per site on that branch *under a strict clock*. But that is wholly different from the number of substitutions per site (as currently labelled) that have happened, or that you would get for a non-clock Bayesian or ML analysis. It is therefore odd to label it as the "number of substitutions". The methods and legend phrasing seems to confound 'Bayesian' with 'Clock-based', and focus on the former (which is basically irrelevant) and ignore the latter (which is dominating the appearance of the tree). More seriously, if a clock has been fitted then it has a root, but the trees are presented unrooted. If they want to force a clock, they should present it as such. As things stand, this is simply making the (probably very noisy) tree look MUCH prettier than it should.

We thank the reviewer for this detailed and technically precise critique. Upon reflection, we agree that the use of a strict molecular clock with a fixed rate of 1 in BEAST introduced methodological ambiguity.

We have therefore re-inferred both phylogenetic trees using maximum likelihood (IQ-TREE), with the substitution model selected automatically by ModelFinder and node support assessed via 1000 ultrafast bootstrap replicates. The RT-domain tree (Fig. 1c) is now rooted using a LINE retrotransposon (I_element) as an outgroup. The insertion tree (Fig. 3c) is presented unrooted, as appropriate for a dataset without a designated outgroup. Branch lengths in both trees now represent true maximum likelihood estimates of substitutions per site under the best-fit model. The methods section, figure legends, and all relevant text have been updated accordingly.

The biological conclusions of the paper are completely untouched: *Kuruka* remains placed within the Gypsy/Gypsy superfamily close to Gypsy5, and the *D. melanogaster* insertions continue to form a well-supported monophyletic clade most closely related to insertions from *D. erecta* and *D. bocqueti*.

Minor point: the majority of the fonts in the majority of the figures are too small.

Where possible, we increased the font to make it more readable.

REVIEWER COMMENTS

The authors did a careful job in addressing the reviewer's comments.

Panel 3b might benefit from some more labelling (marker size, expected size of PCR product, etc.)

We incorporated into the figure the markers size and the expected size of the PCR product.

Overall, I consider the manuscript ready for publication.

congrats on an interesting story.
